# Pulmonary challenge with carbon nanoparticles induces a dose-dependent increase in circulating leukocytes in healthy males

**DOI:** 10.1186/s12890-017-0463-x

**Published:** 2017-09-06

**Authors:** Marieke Berger, Johannes D. de Boer, René Lutter, Michiel Makkee, Peter J. Sterk, Elles M. Kemper, Jaring S. van der Zee

**Affiliations:** 1 Department of Respiratory Medicine, Academic Medical Center, University of Amsterdam, Room F-5-260, Amsterdam, The Netherlands; 2 Center for Experimental and Molecular Medicine (CEMM), Academic Medical Center, University of Amsterdam, Amsterdam, The Netherlands; 3 Department of Experimental Immunology, Academic Medical Center, University of Amsterdam, Amsterdam, The Netherlands; 4 Catalysis Engineering, Chemical Engineering, Technical University of Delft, Delft, The Netherlands; 5 Department of Pharmacy, Academic Medical Center, University of Amsterdam, Amsterdam, The Netherlands; 6 Department of Respiratory Medicine, Onze Lieve Vrouwe Gasthuis, Amsterdam, The Netherlands

**Keywords:** Air pollution, Coagulation, Bronchial provocation test, Bronchoalveolar lavage, Ultrafine particles, Inflammation

## Abstract

**Background:**

Inhalation of particulate matter, as part of air pollution, is associated with increased morbidity and mortality. Nanoparticles (< 100 nm) are likely candidates for triggering inflammatory responses and activation of coagulation pathways because of their ability to enter lung cells and pass bronchial mucosa. We tested the hypothesis that bronchial segmental instillation of carbon nanoparticles causes inflammation and activation of coagulation pathways in healthy humans in vivo.

**Methods:**

This was an investigator-initiated, randomized controlled, dose-escalation study in 26 healthy males. Participants received saline (control) in one lung segment and saline (placebo) or carbon nanoparticles 10 μg, 50 μg, or 100 μg in the contra-lateral lung. Six hours later, blood and bronchoalveolar lavage fluid (BALF) was collected for inflammation and coagulation parameters.

**Results:**

There was a significant dose-dependent increase in blood neutrophils (*p* = 0.046) after challenge with carbon nanoparticles. The individual top-dose of 100 μg showed a significant (*p* = 0.05) increase in terms of percentage neutrophils in blood as compared to placebo.

**Conclusions:**

This study shows a dose-dependent effect of bronchial segmental challenge with carbon nanoparticles on circulating neutrophils of healthy volunteers. This suggests that nanoparticles in the respiratory tract induce systemic inflammation.

**Trial registration:**

Dutch Trial Register no. 2976. 11 July 2011. http://www.trialregister.nl/trialreg/admin/rctview.asp?TC=2976

**Electronic supplementary material:**

The online version of this article (10.1186/s12890-017-0463-x) contains supplementary material, which is available to authorized users.

## Background

Particulate matter (PM), as part of air pollution, is a complex mixture, consisting of variably sized carbon particles with different types of molecules adsorbed to them. There is a strong association between exposure to particulate matter and increased morbidity and mortality [1–3]. However, it is unclear which components of PM are responsible for these health effects. Human inhalation studies showed that exposure to particulate matter from air pollution causes pulmonary and systemic inflammation [4, 5], as measured by blood neutrophils [6], and C-reactive protein, increased thrombogenesis [7] and altered autonomic function, as represented by an increase in blood pressure and heart rate [8].

Nanoparticles, with a diameter of less than 0.1 μm, are likely candidates for causing the pulmonary and systemic effects associated with particulate matter [9, 10], because of their higher oxidant capacity compared to larger particles [11], their higher deposition efficiency in the pulmonary region [12], and their ability to penetrate lung cells [13, 14]. Nanoparticles, while constituting a small fraction of the total mass of ambient particulate matter, represent a major proportion in terms of particle number and surface area [15].

Most human in vivo studies used inhalation challenges with larger particles or complete diesel exhaust, which is composed of different particles with respect to size and arrangement. Although this gives very relevant information about the general health effects of ambient exposures, it still needs to be examined which specific fractions of particulate matter are causatively driving the observed health effects. This is even more important for people exposed to high concentrations of particulate matter on a daily basis due to their occupation, such as tunnel workers or Carbon Black production workers. In order to evaluate the health effects of such extensive, variate exposures, we suggest a systematical, step-by-step approach. Therefore, in the current study we examined the effect of pure, graphitic, onion-like, carbon nanoparticles, and the core fraction of ambient particulate matter, on local (lung) and systemic inflammation and activation of coagulation in human beings.

Previous human in vivo studies focusing on nanoparticles have examined translocation to the systemic circulation [16], and vascular function [17] after whole lung inhalation. In addition, nanotoxicity in experimental animals and in vitro studies appears to be related to several cellular mechanisms, including oxidative stress formation [5, 18], and increase of cytosolic calcium concentration in platelets [19]].

We hypothesized that carbon nanoparticles cause dose-dependent local and systemic activation of inflammatory and coagulation pathways after pulmonary instillation. The aim of the study was to test this hypothesis in humans in vivo by using the well-established method of bronchial segmental challenge [20, 21] in order to avoid serious adverse events.

## Methods

### Subjects

Twenty-six healthy, non-smoking males between 18 and 45 years of age were recruited by advertising. Subjects were included if there were no significant findings during screening, consisting of a medical history, physical examination, lung function measurement (FEV_1_) [22], and hematological and biochemical screening. Volunteers were excluded when having a history of pulmonary disease, enhanced bleeding tendency, or smoking within the past 12 months and more than 5 pack years of smoking history. We continued recruitment until 26 subjects completed all visits, yielding sufficient material for analysis. All volunteers gave written informed consent and the institutional Ethics Committee approved the study (Dutch Trial Register no. 2976. http://www.trialregister.nl/trialreg/admin/rctview.asp?TC=2976).

### Study design

**Fig. 1:**
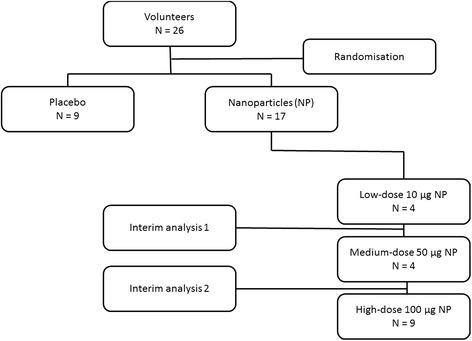
Flow-chart study design

This was an investigator initiated, randomized controlled, single-center, single-blinded dose-escalation study. In order to ensure the safety of the study participants, dose-escalation was performed according to the European Medicines Agency (EMA) First-In-Man (FIM) guidelines [23]. Escalating dosages of 10 μg, 50 μg, and 100 μg of carbon nanoparticles were instilled, by using different groups of volunteers per dose (Fig. 1). Dosages were aimed to be in line with real life exposure concentrations. They were calculated according to the European Medicines Agency (EMA) First-In-Man (FIM) guidelines [23]. Based on the No Observed Adverse Effect Level (NOAEL) in non-clinical safety studies, safe dosages for human use were calculated by adjustment for allometric factors (body volume, surface area). These dosages are identical to measured concentrations at various locations in the Netherlands by Strak et al. [24]. These locations comprised an underground train station and several traffic sites. As this was the first study investigating segmental instillation of the lung with carbon particles, a very low starting dose of 10 μg was chosen. To guarantee the safety of study participants a data safety monitoring board (DSMB) was appointed and after completion of each dose, an interim analysis was performed by this board regarding adverse events, symptoms and white blood cell count in bronchoalveolar lavage fluid.

If any adverse event occurred, the previous dose would be regarded as maximum tolerable dose (MTD), and this dosage group would be completed up to 9 volunteers. Also, if there would be a mean difference of 50% or more in BAL leukocytes (control versus challenge lung), this dosage was to be regarded as minimum effective dose (MED) for this model and this group was to be completed up to 9 volunteers.

### Study day

Healthy males were randomized (by envelopes) to receive bronchial segmental challenge with placebo (20 ml sodium chloride 0.9%) or a suspension of carbon nanoparticles in saline (20 ml sodium chloride 0.9%) in a subsegment of the lingula or right middle lobe. This was performed after control challenge (sodium chloride 0.9%) in the contralateral lung subsegment. Six hours later, a second bronchoscopy with bronchoalveolar lavage (8 times 20 ml) of the challenged segment (nanoparticles or saline) and the saline challenged control segment was performed according to the BTS-guidelines [25]. Both bronchoscopies were preceded by lung function test (FEV_1_) and blood draw, while vital signs were measured regularly during the study day.

### Materials

Carbon nanoparticles (Printex-U) were purchased from Evonik Carbon Black GmbH, Hanau, Germany. The properties of the particles (raw material) and the final product (particles suspended in saline) were thoroughly analyzed for size distribution, arrangement (onion-like), pH, purity (no surface group), contamination with endotoxins, and stability by transmission electron microscopy (TEM), element analysis, and nanoparticle tracking analysis. Analyses were performed by experienced scientists of the Technical University Delft, the Netherlands. This information was registered in a Product Dossier (See Additional file 1), which was presented to the Ethics Committee for assessment prior to the start of the study [26].

For each study participant a fresh suspension (within 12 h of administration) was prepared in a laminar flow cabinet. The carbon powder was accurately weighed and mixed with pulverized sodium chloride by serial dilution technique. A precisely weighed amount of this mixture was suspended in water for injections and the final suspension was sterilized in a validated autoclave (121 °C, 15 min). Endotoxin concentration of the nanoparticle suspension was < 0.01 EU/ml. Before bronchial segmental challenge the nanoparticle suspension was sonicated for 5 min in a table water sonicator.

### Measurements

Spirometry (MasterscreenPneumo; Jaeger; Würzburg, Germany) was performed according to the guidelines of the European Respiratory Society (ERS) [22] and hematological and biochemical analyses on peripheral blood were done in a standardized way by the institutional laboratory of clinical chemistry.

Blood samples were drawn in standard tubes with heparin, EDTA, and citrate. To inhibit proteolytic activity and degradation of complement products we used stabilyte tubes with benzamine (BioPool® Stabilyte™). They were centrifuged at 8^o^ C and 3000 rpm for 10 min in a table centrifuge. Supernatant was stored at −80 °C. BALF fractions 2–8 were pooled (per lung segment) and centrifuged at 8 °C and 1240 rpm for 10 min. Before centrifugation, benzamidine was added to a separate aliquot of BALF. Supernatants were stored at −80°Celcius until we analyzed them in parallel to reduce inter-assay variation. For coagulation measurements, citrate plasma and BALF samples were snap frozen in liquid nitrogen before storage.

In peripheral blood we measured leukocyte cell counts (× 10^9^/L), Cell differentials (× 10^9^/L), C-reactive protein (CRP) in mg/L, von Willebrand Factor in % (Elisa, Homemade), Plasmin-Anti-Plasmin (PAP) in μg/L (Elisa, DRG Diagnostica), D-dimer in mg/L FEU (BCS-XP, Siemens), Endogenous Thrombin Potential (ETP) in % (Calibrated Automated Thrombogram), and TAT complexes in μg/L (Elisa, Siemens, Marburg, Germany).

Cell differentials of 500 cells from BALF were performed on cytospins stained with a modified Giemsa stain (Diff-Quick; Dade Behring AG, Düdingen, Switzerland). Cell concentrations were calculated as (% cells x total cell count)/volume in BALF.

Next to this, we measured local inflammatory cytokines and chemokines in BALF. This comprised Eosinophil Cationic Protein (ECP) and Myeloperoxidase (MPO) by ELISA (Diagnostics Development and Duoset DY3174 R&D, respectively). Interleukin (IL) 6, IL-8, IL-10, IL-17A, IP-10, GRO-α, MCP-1, MIP-1α, MIP-1β, Tumor Necrosis Factor alpha (TNF-α), and Vascular Endothelial Growth Factor (VEGF) were determined by multiplex bead flow assays (BioRad) and read on a Bioplex 200 reader (Bio-Rad Laboratories, Inc., Hercules, CA). Concerning coagulation parameters, we measured D-dimer (Elisa, Stago Diagnostica), Plasminogen-Activator-Inhibitor-Antigen (PAI-Ag) (Elisa, BioMed), and TAT complexes (Elisa, Siemens, Marburg, Germany). All measurements were performed by experienced and qualified technicians who were blinded to the clinical details.

In order to determine the quality of assay performance, we calculated the inter-assay coefficients of variation (CV). For the primary outcome parameters (white blood cell counts), the inter-assay CV’s for total leucocytes, neutrophils, lymphocytes and eosinophils are 1,9%, 2,5%, 3,7% and 9,2% respectively.

Concerning the secondary outcome parameters, only ECP and MPO were analyzed by ELISA. The inter-assay CV’s were 13% and 14% respectively.

Other secondary outcomes were analyzed by multiplex bead flow assays. For these assays, we checked with the controls to determine whether the cytokines were properly measured and the software calculated the upper and lower limit of quantification (ULOQ and LLOQ).

### Statistical analysis

The primary endpoint was total leukocyte cell count and differentials in peripheral blood and BALF, while secondary endpoints comprised safety parameters and other markers of inflammation and coagulation activation in peripheral blood and BALF including activation of cytokine/chemokine networks, complement activation, and activation of the protein C system.

Based on previous studies with LPS challenge using the same research protocol (20;28) a sample size of 18 (placebo versus highest dosage nanoparticles) was estimated to have a power of 80% to detect a 50% difference in BALF leukocytes between placebo (saline) and nanoparticle challenged lung segments. *P*-values less than 0.05 were considered significant.

For the dose-response evaluation of blood parameters we performed non-parametric Spearman correlation test of the dose versus the change in blood values before challenge and 6 h after challenge.

We analyzed blood and BALF parameters of the placebo group and the subjects who received the top dose of 100 μg nanoparticles with non-parametric Mann-Whitney test. All analyses were performed with SPSS 20 for Windows.

## Results

Subject characteristics were not significantly different between groups (*p* > 0.05, Table 1). From 26 screened and randomized participants, one did not complete the second bronchoscopy due to medical reasons (unrelated to the study). We were able to collect blood samples before and 6 h after provocation from 26 patients. Analysis of BALF and coagulation parameters in blood was performed in 25 subjects.

**Table 1:** Baseline characteristics

	Placebo (*n* = 9)	10 μg NP (*n* = 4)	50 μg NP (*n* = 4)	100 μg NP (*n* = 9)	*P*-value
Age, Year^a^	26.0 (22–30)	26.0 (26–27)	21.5 (20–31)	24.0 (19–35)	0.70
FEV_1_, Liter^b^	4.92 (0.70)	5.23 (1.19)	4.55 (0.37)	5.05 (0.95)	0.78
Blood leukocytes, Cells × 10^9^/L^b^	5.43 (1.16)	5.70 (0.52)	5.10 (0.62)	5.70 (1.60)	0.85

Values are expressed as ^a^Median (Range), ^b^Mean ± SD, and analyzed by One-Way ANOVA. *Abbreviations*: *NP* nanoparticles, *FEV*_*1*_ forced expiratory volume in 1 s

### Safety analysis

Interim analysis, as assessed by the DSMB, after completion of each dose-group showed no significant adverse events, symptoms, or doubling of white blood cell counts. There was one participant, who received 10 μg carbon nanoparticles, who had complaints of fever and chest pain after the study day. He showed no signs of pneumonia, pneumothorax or pulmonary embolism. The subject was treated with analgesics for one day, after which the complaints disappeared.

### Inflammation parameters: *Dose-response correlations*

**Fig. 2:**
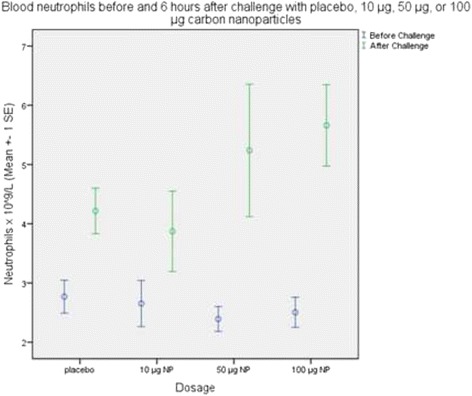
Peripheral blood neutrophils before (black bars) and 6 h after (green bars) bronchial segmental challenge with placebo, 10 μg, 50 μg, or 100 μg carbon nanoparticles (NP). Values are expressed as mean ± 1 Standard Error

There was a significant dose-dependent increase in blood neutrophils (Spearman *p* = 0.0468) and a trend towards increased blood leukocytes (*p* = 0.061) after challenge with carbon nanoparticles as compared to placebo. Figure 2 shows the means of circulating neutrophils before and 6 h after bronchial segmental challenge with placebo, 10 μg, 50 μg, and 100 μg carbon nanoparticles. In Table 2 the means of circulating leukocytes and neutrophils are shown after segmental challenge. In BALF there were no significant differences measured between the different dosage groups. Additionally, we compared the results of the different groups with each other. Additional file 2: Table S1 shows the analysis of the inflammation parameters after challenge with placebo as compared to 10 μg nanoparticles in blood and BALF. In Table S2 of the Additional file 2 the results of the comparison between placebo and 50 μg nanoparticles is shown. Results of the analysis between 10 μg nanoparticles and 50 μg nanoparticles are described in Additional file 2: Table S3. Comparison of 10 μg nanoparticles versus 100 μg nanoparticles is shown in Additional file 2: Table S4. Analysis of 50 μg nanoparticles as compared to the 100 μg nanoparticle group is presented in Table S5 of the Additional file 2.

**Table 2:** Inflammation parameters in blood 6 h after bronchial segmental challenge with placebo, 10 μg, 50 μg, or 100 μg carbon nanoparticles

Peripheral Blood Cells × 10^9^/L	Placebo (*n* = 9)	10 μg NP (*n* = 4)	50 μg NP (*n* = 4)	100 μg NP (*n* = 9)	*P*-value
Leukocytes	6.61 ± 0.38	6.10 ± 0.45	7.65 ± 1.13	8.24 ± 0.77	0.061
Neutrophils	4.22 ± 0.38	3.87 ± 0.68	5.24 ± 1.12	5.66 ± 0.69	0.046
Lymphocytes	1.76 ± 0.11	1.64 ± 0.28	1.78 ± 0.17	1.89 ± 0.11	0.934
Monocytes	0.49 ± 0.03	0.48 ± 0.04	0.55 ± 0.08	0.57 ± 0.06	0.156
Eosinophils	0.13 ± 0.03	0.07 ± 0.03	0.07 ± 0.03	0.10 ± 0.04	0.602
Basophils	0.03 ± 0.01	0.04 ± 0.01	0.03 ± 0.01	0.02 ± 0.00	0.970

Values are expressed as mean ± SEM. The correlations between dose of nanoparticles and changes from baseline of cell numbers were non-parametrically analyzed by Spearman correlation. *Abbreviations*: *NP* nanoparticles

### Inflammation parameters: *Placebo* versus *100* μg *Nanoparticles* (non-parametric analysis, table 3)

Segmental challenge with the individual top-dose of 100 μg carbon nanoparticles showed a significant relative increase of neutrophils (*p* = 0.05) in peripheral blood as compared to placebo (See Table 3). No other significant differences in circulating inflammation parameters were observed between these groups.

**Table 3:** Inflammation parameters in blood and BALF 6 h after bronchial segmental challenge with placebo or 100 μg nanoparticles

Inflammation Parameters Blood	Placebo *N* = 9	100 μg NP *N* = 9	*P*-value
Leukocytes, Cells × 10^9^/L	6.60 (4.8–8.5)	8.70 (5.6–12.4)	0.161
Neutrophils, Cells × 10^9^/L	4.35 (2.9–6.6)	6.15 (3.4–9.7)	0.050
Lymphocytes, Cells × 10^9^/L	1.76 (1.4–2.3)	1.77 (1.5–2.5)	0.931
Monocytes, Cells × 10^9^/L	0.52 (0.3–0.6)	0.59 (0.3–0.9)	0.387
Eosinophils, Cells × 10^9^/L	0.11 (0.1–0.3)	0.07 (0.0–0.4)	0.931
Basophils, Cells × 10^9^/L	0.03 (0.0–0.1)	0.02 (0.0–0.1)	0.931
CRP, mg/L	0.50 (0.3–7.4)	0.40 (0.3–3.5)	1.000
Inflammation Parameters BALF	Placebo *N* = 8	100 μg NP *N* = 9	*P*-value
Leukocytes, Cells × 10^4^/ml	9.47 (6.5–36.5)	11.63 (5.7–18.7)	1.000
Neutrophils, Cells × 10^4^/ml	0.33 (0.1–2.6)	0.58 (0.1–3.8)	0.637
Lymphocytes, Cells × 10^4^/ml	0.31 (0.1–0.7)	0.40 (0.1–1.6)	0.153
Monocytes, Cells × 10^4^/ml	-	-	1.000
Eosinophils, Cells × 10^4^/ml	0.03 (0.0–0.3)	0.00 (0.0–0.1)	0.637
Macrophages, Cells × 10^4^/ml	8.72 (6.0–33.1)	9.04 (5.0–16.9)	0.347
Mast cells, Cells × 10^4^/ml	0.00 (0.0–0.0)	0.00 (0.0–0.0)	--
Plasma cells, Cells × 10^4^/ml	-	-	--
ECP, ng/ml	0.65 (0.3–2.3)	0.76 (0.1–1.9)	0.347
MPO, ng/ml	13.91 (5.1–43.3)	14.28 (6.3–35.7)	1.000
GRO-α, ng/ml	0.60 (0.3–0.9)	0.49 (0.2–1.5)	1.000
IL-6, pg/ml	5.39 (1.0–57.0)	25.57 (0.2–124.0)	1.000
IL-8, ng/ml	0.03 (0.0–0.5)	0.13 (0.0–2.2)	1.000
IL-17, pg/ml	1.60 (1.6–1.6)	1.60 (1.6–1.6)	--
CXCL-10, ng/ml	0.66 (0.1–5.6)	1.02 (0.1–5.3)	1.000
MCP-1, ng/ml	0.02 (0.0–0.1)	0.02 (0.0–0.8)	1.000
MIP-1α, pg/ml	0.15 (0.2–8.5)	3.76 (0.2–31.1)	1.000
MIP-1β, ng/ml	0.06 (0.0–1.0)	0.48 (0.0–7.1)	1.000
TNF-α, pg/ml	0.60 (0.6–1.5)	1.15 (0.6–15.1)	0.576
VEGF, ng/ml	0.78 (0.0–1.7)	0.51 (0.2–1.7)	0.057

Values are expressed as median (minimum –maximum values). Changes in blood values (pre-challenge baseline and 6 h post-challenge) and BALF values (control segment and challenged segment 6 h after challenge) were non-parametrically analyzed by Mann-Whitney test

*Abbreviations*: *NP* nanoparticles, *CRP* C-reactive protein, *ECP* Eosinophil Cationic Protein, *MPO* Myeloperoxidase, *GRO-α* Growth Regulated Oncogene-alpha, *IL* Interleukin, *CXCL10* Interferon gamma-induced protein 10, *MCP-1* Monocyte Chemoattractant Protein 1, *MIP-1α* Macrophage Inflammatory Protein 1 alpha, *MIP-1β* Macrophage Inflammatory Protein 1 beta, *TNF-α* Tumor Necrosis Factor alpha, *VEGF* Vascular Endothelial Growth Factor

Data on the analysis between the different dosage groups is presented in the Additional file 2. Measurements of monocytes, mast cells, plasma cells, and IL-17A were below the detection limit.

### Coagulation parameters: *Placebo* versus *100* μg *Nanoparticles (Non-parametric analysis)*

Comparison of coagulation parameters after bronchial segmental challenge with saline (placebo) or 100 μg nanoparticles showed no significant differences between those groups. Table 4 summarizes the coagulation parameters in blood and BALF after bronchial segmental challenge with placebo or 100 μg carbon nanoparticles.

**Table 4:** Coagulation parameters in blood and BALF 6 h after bronchial segmental challenge with placebo or 100 μg nanoparticles

Coagulation Parameters In Blood	Placebo *N* = 8	100 μg NP *N* = 9	*P*-value
vWF, In percentage	80.50 (19.0–149.0)	95.00 (53.0–115.0)	1.000
TATc, in μg/L	2.85 (2.3–26.6)	2.70 (2.3–3.3)	0.347
D-dimer, in mg/L FEU	0.17 (0.2–0.2)	0.17 (0.2–0.2)	1.000
PAP, In mg/L	0.68 (0.3–1.4)	0.46 (0.4–0.6)	0.347
ETP, in percentage	93.00 (85.0–117.0)	98.00 (79.0–103.0)	1.000
Coagulation parameters in BALF	Placebo *N* = 8	100 μg NP *N* = 9	*P*-value
TATc, in μg/L	2.85 (1.1–10.9)	2.80 (0.1–8.8)	0.347
D-dimer, in μg/L	7.00 (2.0–17.0)	5.00 (1.0–29.0)	0.050
PAI-Ag, in ng/mL	0.50 (−0.5–0.5)	0.50 (0.5–0.5)	-

Values are expressed as median (minimum – maximum values). Changes in blood values (pre-challenge baseline and 6 h post-challenge) and BALF values (control segment and challenged segment 6 h after challenge) were non parametrically analyzed by Mann-Whitney test

*Abbreviations*: *NP* nanoparticles, *vWF* von Willebrand Factor, *TATc* Thrombin AntiThrombin Complex, *PAP* Plasmin-Anti-Plasmin, *ETP* Endogenous Thrombin Potential, *PAI-Ag* Plasminogen-Activator-Inhibitor-Antigen

## Discussion

This study shows that bronchial segmental challenge with carbon nanoparticles up to a maximum of 100 μg is safe, and causes a significant dose-dependent increase in circulating neutrophils and a trend towards increased leukocytes. In the subgroup of patients challenged with 100 μg nanoparticles, there was a significant relative increase of neutrophils in peripheral blood as compared to placebo (non-parametric analysis by Mann Whitney test). These results suggest the activation of an acute phase response after challenge with carbon nanoparticles.

To our knowledge, this is the first study investigating the effect of bronchial segmental challenge with carbon nanoparticles on local and systemic inflammation and coagulation. The study is the first part of a stepwise approach, examining which characteristics and constituents of air pollution can be responsible for the observed health effects. We observed a significant dose-dependent effect on circulating neutrophils. This seems to be in line with previous studies investigating human inhalation of diesel exhaust, air pollution, and nanoparticles [6, 27, 28], who showed a significant increase in circulating cells and interleukins. Interestingly, Frampton and colleagues [28], showed a reduced expression of adhesion molecules on blood leukocytes after inhalation of generated ultrafine elemental carbon particles during exercise. This might be caused by an effect of nanoparticles on the pulmonary vasculature. Concerning coagulation parameters, we found no differences between placebo and 100 μg nanoparticle challenge. This is in contrast with Viehmann et al. [7], who found associations between long-term exposure to fine particulate matter and increased high-sensitivity C-reactive protein and platelets.

Taken together, our data extend previous observations of inflammation into the nanoparticle range.

We believe that bronchial segmental challenge can be regarded as a strength of the present study. Previous investigators showed that it is a safe, and well-tolerated technique [20, 21, 29], in which a limited amount of lung tissue is exposed to the challenging agent, reducing the risk of bronchoconstriction or an allergic reaction. Another advantage for measuring local effects by this research model is that a placebo challenge in a subsegment of the contra-lateral lung can be used as a control during the same experiment in the same subject**.** Therefore, we were able to reduce the amount of participants. In spite of the safety and advantages of this research model, there are still risks related to the procedure of bronchoscopy. For instance, it could be possible that segmental instillation causes local airway injury due to the relatively high concentration of particles to a small surface area. In this study, we only observed small differences between placebo and nanoparticle challenges. Therefore, we think that the possible local airway injury at the site of instillation is caused by instillation in general and not dependent of the amount of nanoparticles in the suspension. Nevertheless, we should emphasize that real life exposure is by inhalation and not by instillation.

In order to increase safety, we used a dose-escalation design, and small dosages of nanoparticles similar to real life exposures in the Netherlands, as observed by Strak et al. [24]. The dosages were also comparable with several animal studies [30, 31]. Next to this, we selected neutral, apolar, spherical, and pure carbon nanoparticles which resemble the carbon particles in air pollution concerning particle characteristics. Finally, we performed an extensive characterization of the carbon nanoparticles by building a Product Dossier (See Additional file 1) [26].

Nevertheless, the current study also has a few limitations. We tried to prevent the particles from clustering by ultrasonification. Although the majority of particles were representing separate nanoparticles as shown by transmission electron microscopy (TEM), we could not avoid the presence of some larger agglomerates in the final nanoparticle suspension in saline. This contrasts to Creutzenberg et al., who showed no change in diameter of carbon black agglomerates after instillation in rats [30].

Secondly, we powered on local and systemic inflammation parameters, using blood and BALF leukocyte counts as primary outcome. Nevertheless, considering our results, which show a significant effect on circulating neutrophils in the subgroup challenged with 100 μg nanoparticles, we cannot exclude that the study is underpowered with respect to the individual top-dose of 100 μg nanoparticles.

Furthermore, the time-frame of our study, in which we collected the blood and BALF 6 h after the provocation, covers the early phase of the inflammatory response. We may have missed possible effects of carbon nanoparticles on inflammation or coagulation within these 6 h or later. As shown by Gardner et al. [32]*,* the increased risk of myocardial infarction after exposure to elevated concentrations of air pollution varies from one hour up to a few days. Also, we performed the challenges in healthy males and we don’t know whether the results can be extrapolated to females.

The underlying mechanisms of how carbon nanoparticles can induce the systemic increase in neutrophils is as yet unknown. Saber and colleagues [15], propose that nanoparticles induce a strong pulmonary acute phase response in which *Saa3* is upregulated, causing neutrophil influx into the lungs. Whereas Franklin et al. [8] hypothesize that spillover of inflammatory mediators and cells from the lungs into the systemic circulation causes the systemic effects of inhalation of nanoparticles, our data do not favor the latter explanation, as we observed only limited local inflammatory responses to nanoparticles in BALF.

Animal and in vitro models have also shown that nanoparticle inhalation or instillation can induce an acute inflammatory response [33]. Observed mechanisms concern oxidative stress [5, 18], epithelial damage [34], polarization of Th17 leading to enhanced differentiation [35], and an increase of cytosolic calcium concentration in platelets [19]. However, these effects are not specific for carbon nanoparticles.

We observed no significant changes in coagulation related parameters. This is in line with previous human inhalation studies which showed inconclusive results concerning the thrombotic tendency of diesel exhaust as measured by CRP, von Willebrand Factor, PAI-1, and platelets [15, 36].

Notably, our data show an increase in circulating neutrophils after challenge with low dose, pure, and clean carbon nanoparticles. C-reactive protein was not increased 6 h after challenge, probably due to a more delayed response time as compared to circulating neutrophils. These findings seem to be relevant, since increased circulating inflammatory parameters, such white blood cell count (WBC), neutrophils and CRP are associated with an increased cardiovascular risk, and a higher mortality [15, 37, 38].

It should be emphasized that the present study was done in healthy volunteers. We think it is not unlikely that more susceptible subjects with pre-existing morbidity, such as asthma [39], COPD, cystic fibrosis [40], or vascular disease, are experiencing more intense acute biological effects after exposure to carbon nanoparticles. In addition, it should be noted that the present study merely addressed acute effects of short-term carbon nanoparticle exposure. Therefore, it cannot be excluded that long-term, repeated exposures in real-life situations, such as experienced by tunnel workers and industrial workers [41], can have more impact on inflammatory and coagulant pathways. Finally, it is likely that other parts of particulate matter, such as oxidized particles are at least in part responsible for the health effects described. The present data warrant further studies to address these issues.

## Conclusions

In conclusion, we showed that bronchial segmental instillation of pure, apolar, and neutral carbon nanoparticles, is a safe and effective research model. We observed a small, dose-dependent increase in blood leukocytes and blood neutrophils after we challenged healthy males with low dosages of carbon nanoparticles as compared to the daily exposure concentration of certain occupational groups. These results may point towards a pathophysiologic background for the observed health risks associated with increases in air pollution, and merit the next research steps to identify the causative agents.

## Additional files

Additional file 1: Product Dossier. Carbon black nanoparticles (Printex-U). (DOC 3786 kb)

Additional file 2: Table S1. Inflammation parameters in blood and BALF after challenge with placebo or 10 μg nanoparticles. Values are expressed as median with minimum and maximums values. Analysis was performed with non-parametric Mann - Whitney test, after correction for baseline values. *Definition of abbreviations: NP: nanoparticles, CRP: C-reactive protein*. **Table S2.** Inflammation parameters in blood and BALF after challenge with placebo or 50 μg nanoparticles. Values are expressed as median with minimum and maximum values. Analysis was performed with non-parametric Mann - Whitney test, after correction for baseline values. *Definition of abbreviations: NP: nanoparticles, CRP: C-reactive protein*. **Table S3.** Inflammation parameters in blood and BALF after challenge with 10 μg or 50 μg nanoparticles. Values are expressed as median with minimum and maximum values. Analysis was performed with non-parametric Mann - Whitney test, after correction for baseline values. *Definition of abbreviations: NP: nanoparticles, CRP: C-reactive protein*. **Table S4.** Inflammation parameters in blood and BALF after challenge with 10 μg or 100 μg nanoparticles. Values are expressed as median with minimum and maximum values. Analysis was performed with non-parametric Mann - Whitney test, after correction for baseline values. *Definition of abbreviations: NP: nanoparticles, CRP: C-reactive protein*. **Table S5.** Inflammation parameters in blood and BALF after challenge with 50 μg or 100 μg nanoparticles. Values are expressed as median with minimum and maximum values. Analysis was performed with non-parametric Mann - Whitney test, after correction for baseline values. *Definition of abbreviations: NP: nanoparticles, CRP: C-reactive protein*. (DOC 193 kb)

## Abbreviations

ANOVA: ANalysis Of VAriance
ATS: American Thoracic Society
BAL(F): BronchoAlveolar Lavage (Fluid)
CRP: C-reactive protein
CXCL10: Interferon gamma-induced protein 10
DSMB: Data safety monitoring board
ECP: Eosinophil Cationic Protein
EMA: European Medicines Agency
ERS: European Respiratory Society
ETP: Endogenous Thrombin Potential
FEV_1_: Forced expiratory volume in 1 s
FIM: First-In-Man
GINA: Global INitiative for Asthma
GRO-α: Growth Regulated Oncogene-alpha
IL: Interleukin
MCP-1: Monocyte Chemoattractant Protein 1
MIP-1α: Macrophage Inflammatory Protein 1 alpha
MIP-1β: Macrophage Inflammatory Protein 1 beta
MPO: Myeloperoxidase
NOAEL: No Observed Adverse Effect Level
PAI-Ag: Plasminogen-Activator-Inhibitor-Antigen
PAP: Plasmin-Anti-Plasmin
PM: Particulate matter
TAT: Thrombin-AntiThrombin
TNF-α: Tumor Necrosis Factor alpha
VEGF: Vascular Endothelial Growth Factor
WBC: White blood cell count
