# Supplementary material for: Pulmonary challenge with carbon nanoparticles induces a dose-dependent increase in circulating leukocytes in healthy males
Source: BMC Pulm Med. 2017 Sep 6;17:121. doi: 10.1186/s12890-017-0463-x (PMC5588713; doi:10.1186/s12890-017-0463-x)
Supplement: Supplementary file 1 — Product Dossier. Carbon black nanoparticles (Printex-U). (DOC 3786 kb) [file 12890_2017_463_MOESM1_ESM.doc]

Additional file 1

product dossier

Carbon Black nanoparticles (printex-u), VERSION 2
21^th^ of november 2011

AUTHORS

| M. Berger, MD | Department of Pulmonology  Academic Medical Center, University of Amsterdam  Meibergdreef 9, Room F5-260, 1105 AZ Amsterdam  Tel+31205668375, Fax +31205669001  E-mail: [m.berger@amc.uva.nl](mailto:m.berger@amc.uva.nl) |
| --- | --- |
| Dr E.M. Kemper, PhD Hospital Pharmacist and Clinical Pharmacologist | Institutional Pharmacy  Academic Medical Center, University of Amsterdam  Meibergdreef 9, E0, 1105 AZ Amsterdam  Tel: + 31 20 5667955  E-mail: e.m.kemper@amc.uva.nl |
| Dr J. S. van der Zee, MD, PhD | Department of Pulmonology  Academic Medical Center, University of Amsterdam  Meibergdreef 9, 1105 AZ Amsterdam  E-mail: [j.s.vanderzee@amc.uva.nl](mailto:j.s.vanderzee@amc.uva.nl) |

TABLE OF CONTENTS

1. INTRODUCTION

2.1 CHEMICAL PHARMACEUTICAL DATA

2.1.S **SUBSTANCE (Printex-U)**

2.1.S.1 General Information

2.1.S.1.1 Nomenclature

2.1.S.1.2 Structure

2.1.S.1.3 General Properties

2.1.S.2 Manufacture:

2.1.S.2.1 Manufacturer(s)

2.1.S.2.2 Description of Manufacturing Process and Process

2.1.S.2.3 Control of Materials

2.1.S.2.4 Controls of Critical Steps and Intermediates

2.1.S.2.5 Process validation and/or Evaluation

2.1.S.2.6 Manufacturing Process Development

2.1.S.3 Characterisation:

2.1.S.3.1 Elucidation of Structure and Other Characteristics

2.1.S.3.2 Impurities

2.1.S.4 Control of Substance:

2.1.S.4.1 Specification

2.1.S.4.2 Analytical Procedures

2.1.S.4.3 Validation of Analytical Procedures

2.1.S.4.4 Batch Analyses

2.1.S.4.5 Justification of specification

2.1.S.5 Reference Standards or Materials

2.1.S.6 Container Closure System

2.1.S.7 Stability

2.1.P **PRODUCT (Printex-U–suspension in saline)**

2.1.P.1 Description and Composition of the Product

2.1.P.2 Pharmaceutical Development:

2.1.P.2.1 Components of the Product

2.1.P.2.2 Product

2.1.P.2.3 Manufacturing Process Development

2.1.P.2.4 Container Closure System

2.1.P.2.5 Microbiological Attributes

2.1.P.2.6 Compatibility

2.1.P.3 Manufacture:

2.1.P.3.1 Manufacturer(s)

2.1.P.3.2 Batch Formula

2.1.P.3.3 Description of Manufacturing Process and Process Controls

2.1.P.3.4 Controls of Critical Steps and Intermediates

2.1.P.3.5 Process Validation and/or Evaluation

2.1.P.4 Control of Excipients

2.1.P.4.1 Specifications:

2.1.P.4.2 Analytical Procedures

2.1.P.4.3 Validation of Analytical Procedures

2.1.P.4.4 Justification of Specifications

2.1.P.4.5 Excipients of Human or Animal Origin

2.1.P.4.6 Novel Excipients

2.1.P.5 Control of Product:

2.1.P.5.1 Specification(s)

2.1.P.5.2 Analytical Procedures

2.1.P.5.3 Validation of Analytical Procedures

2.1.P.5.4 Batch Analyses

2.1.P.5.5 Characterization on impurities

2.1.P.5.6 Justification of Specification(s)

2.1.P.6 Reference Standards or Materials:

2.1.P.7 Container Closure System:

2.1.P.8 Stability:

2.2 NON-CLINICAL PHARMACOLOGY, PHARMACOKINETICS AND TOXICOLOGY

2.2.1 Test Materials used in Toxicity Studies

2.2.2 Integrated Assessment of the data package

2.2.3 List of studies Conducted & References

2.2.4 GLP statement and Bioanalytical Methods

2.3 CLINICAL DATA

2.3.1 Clinical Pharmacology

2.3.2 Clinical Pharmacokinetics

- - 1. Human Exposure
  1. BENEFITS AND RISKS ASSESSMENT
  2. APPENDICES
     1. APPENDIX I: Material Safety Data Sheet
     2. APPENDIX II: Inspection Certificate
     3. APPENDIX III: Maintenance Certificate TEM
     4. APPENDIX IV: Production Protocol and Labels
     5. APPENDIX V: Sterility process information
     6. APPENDIX VI: Size distribution of product 20 μg/mL
     7. APPENDIX VII: Size distribution of product 200 μg/mL

LIST OF FIGURES

Figure 1: Carbon Black with high mean primary particle size

Figure 2: Degussa Gas Black Process

Figure 3: Transmission Electron Microscopic (TEM) image of Printex-U

Figure 4: Element analysis of Printex-U by Transmission Electron Microscopy - Energy-Dispersive X-ray spectroscopy (TEM-EDX)

Figure 5: Schematic figure of a Transmission Electron Microscope

Figure 6: Horseshoe crab, *Limulus polyphemus*

LIST OF TABLES

Table 1: Specifications for Printex-U

Table 2: Characteristics of Printex-U

Table 3: Justification of specification of Printex-U

##### Table 4: Specifications for Printex-U-suspension in saline

Table 5: Justification of specifications for Printex-U-suspension in saline

CHEMICAL PHARMACEUTICAL AND BIOLOGICAL DATA

Introduction

This Product Dossier presents information relating to a suspension of carbon black nanopowder with Sodium Chloride (NaCl) in sterile water (Aqua dest), which is used as a model for investigating the effects of air pollution on the human lung by bronchial segmental instillation. This is a validated research model by which a small amount of the substance of interest is delivered by a flexible bronchoscope to a subsegment of the lung. The great advantage of this model lies in the reduced exposition to the substance compared to whole lung provocation, and therefore the reduced risk of bronchoconstriction and dyspnea.

Although the suspension of carbon nanopowder is not an Investigational Medicinal Product, we consider it very important for this model to have a qualitatively solid, well analyzed, and sterile product (see Annex 10; Manufacture of pressurized metered dose aerosol preparations for inhalation). Therefore we used the format of IMPD from the CCMO website for this document.

The basis of our final product (suspension of carbon black nanopowder with NaCl in Aqua dest) is Printex-U, which is, like all carbon blacks, the product of incomplete combustion of hydrocarbons.

##### SUBSTANCE

##### General Information

##### Nomenclature

Trade Name: Printex - U Powder

Chemical Name: Carbon Black

CAS Number: 1333-86-4

EG Number: 215-609-9

Chemical Family: Carbon Black

Material No.: 25.1615.0000.94

Production Date: 23.04.2010

Control-/Lot No.: 1660204011

##### Structure

Printex-U is an amorphous carbon powder with a primary particle size of 25nm, and a BET (Brunauer Emmett Teller) Surface Area of about 100m^2^/g.

##### General Properties

##### Dependent on the manufacturing process, a wide range of different Carbon Black Pigment grades with respect to their primary particle size, structure, surface area, and surface chemistry can be produced.

 
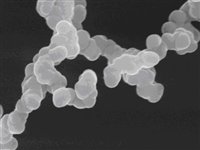


Figure 1: Carbon Black with high mean primary particle size

Particles can be defined as primary particles, aggregates or agglomerates. Primary particles form firmly linked aggregates, which are in turn joining together to form agglomerates. During dispersion in liquid substances these agglomerates are fragmented into aggregates. In table 1 the specifications for Printex-U are described.

Table 1: Specifications for Printex-U

| *Attribute* | *Method* | *Acceptance criteria* |
| --- | --- | --- |
| *Appearance* | *Visual observation* | *Black powder* |
| *Melting point* | *PhEur* | *> 500 °C* |
| *Purity*  *- Heavy metals* | *Element analysis by Energy-dispersive X-ray spectroscopy* | *< 2% of the product* |
| *Water solubility* |  | *in soluble* |
| *pH* |  | *~ 7 (50 g/l, 20°C)* |
| *Flammability* | *VDI 2263* | *> 30 s* |
| *Ignition Temperature* | *VDI 2263 (BAM-furnace)* | *> 200 °C* |
| *Autoinflammability* | *IMDG-Code with 1L sample* | *> 140 °C* |
| *Lower explosion limit* | *VDI 2263* | *Dust 50 g/m^3^* |
| *Upper explosion limit* |  | *Not determined* |
| *Max absolute explosive pressure* | *VDI 2263* | *10 bar* |
| *Vapour pressure* |  | *Not applicable* |
| *Density* | *At 20 °C* | *1,7 – 1,9 g/cm^3^* |
| *Bulk density* |  | *50 – 250 kg/m^3^* |
| *Viscosity, dynamic* |  | *Not applicable* |

Other information:

Dusts can form explosive mixtures with air. However, in this situation, carbon black cannot easily be caused to explode because of the use of small amounts and storage of the substance in a plastic flacon.

#### 2.1.S.2 MANUFACTURE

##### 2.1.S.2.1 Manufacturer(s)

##### Company: Evonik Carbon Black GmbH

##### Produktsicherheit

Postfach 1345, D-63403 Hanau

Telephone +49(0) 6181 59-6013

Telefax +49(0) 6181 59-4205

Email Address sds.asfp@evonik.com

##### 2.1.S.2.2 Description of Manufacturing Process and Process Controls

##### Description of Manufacturing Process

Printex-U, like all carbon blacks is the product of incomplete combustion of hydrocarbons. The most important production methods are the Furnace Black, the Degussa Gas Black, the Lamp Black and the Thermal Black

Printex-U is produced by the Degussa Gas Black production method (figure 2).


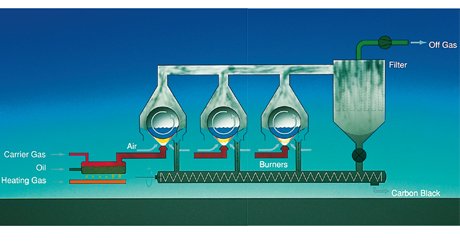


Figure 2: Degussa Gas Black Process

In the Gas Black Process, the raw material – the so-called feedstock - is partially vaporized. The residual oil is continuously withdrawn. The oil vapor is transported to the production apparatus by a combustible carrier gas (e.g. hydrogen, coke oven gas, or methane). Air may be added to the oil-gas mixture for the manufacture of very small particle size carbon black.

A gas black apparatus consist of a burner pipe approximately 5m long, which carries 30-50 diffusion burners. The flames burn in contact with a water-cooled drum, where about half of the formed carbon black is deposited.

##### 2.1.S.2.3 Control of Materials

##### The so-called feedstock originates from natural resources.

##### 2.1.S.2.4 Controls of Critical Steps and Intermediates

Not Applicable

##### 2.1.S.2.5 Process Validation and/or Evaluation

##### Not Applicable

##### 2.1.S.2.6 Manufacturing Process Development

#### Confidential information of the manufacturer

#### 2.1.S.3 CHARACTERISATION OF PRINTEX-U

##### Because the manufacturer is not keen on clinical experiments with their product, they were not willing to give detailed information. To confirm that the product is suitable for our experiments, we performed extensive testing.

Scientists of the section of Catalysis Engineering of the TU-Delft analyzed Printex-U by Transmission Electron Microscopic (TEM) (figure 3) and Energy-Dispersive X-ray spectroscopy (EDX) (figure 4) to determine primary particle size, and exclude the presence of heavy metals.

See Material Safety Data Sheet and Inspection Certificate (Appendices I and II) for characterization by the manufacturer.


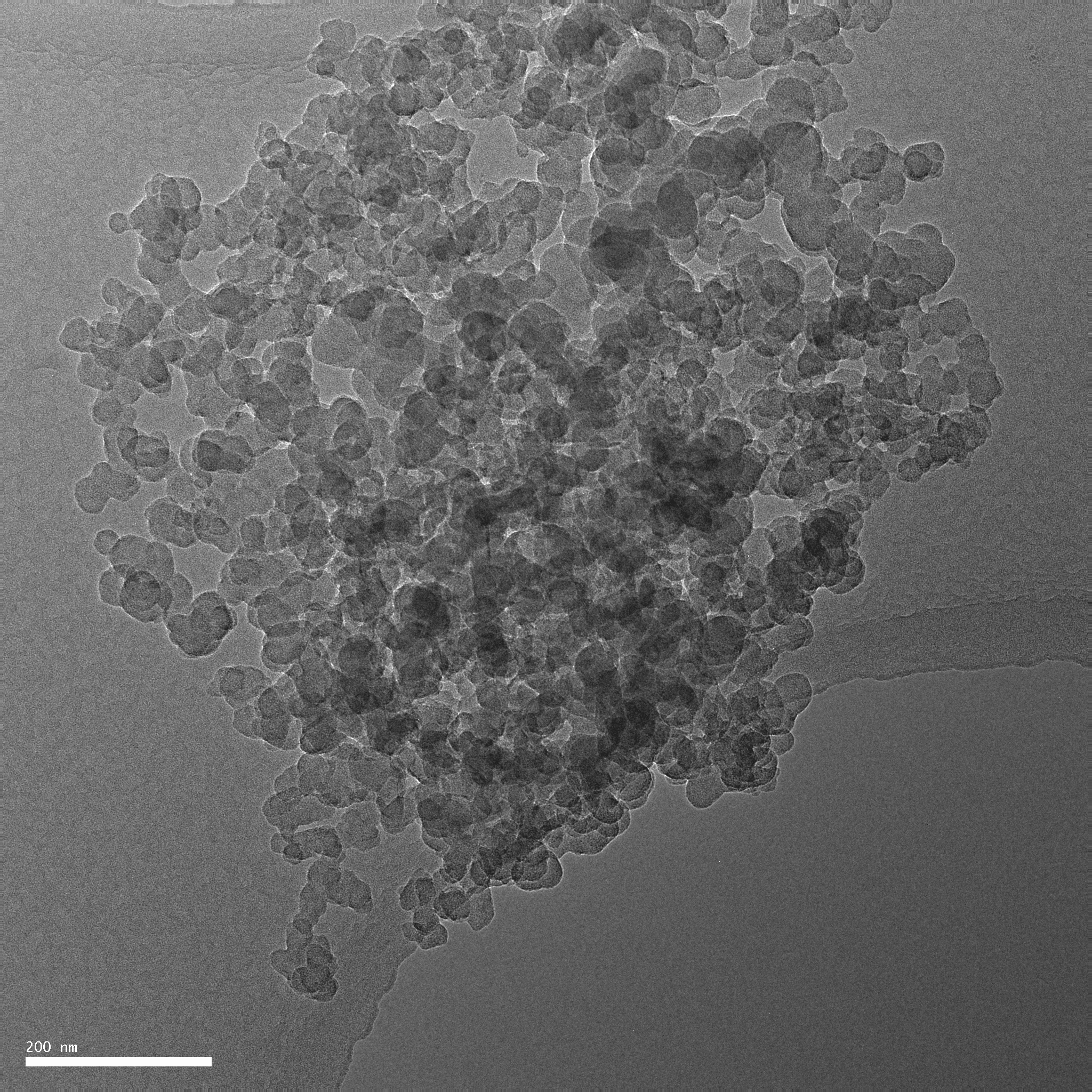


Figure 3: Transmission Electron Microscopic (TEM) image of Printex-U. The scale on the left bottom is 200nm. A cluster of particles with a primary particle size < 50nm is shown


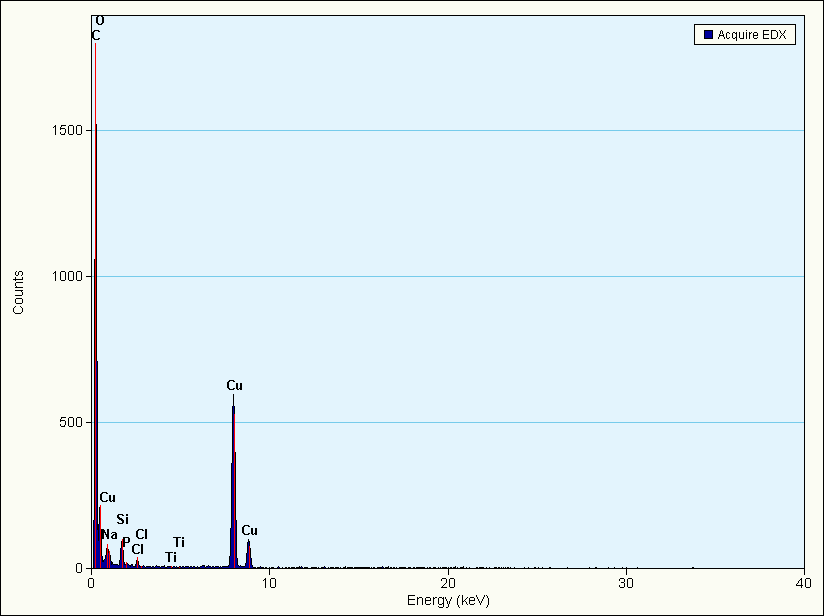


Figure 4: Element analysis of the raw material by Transmission Electron Microscope -Energy-dispersive X-ray spectroscopy (TEM-EDX). The carbon peak is visible on the left at 0.4 keV with a count of 1825. *Note:* the copper peak (Cu) comes from the grid on which the particles are deposited for measurement in the transmission electron microscope (TEM). Considering the size of the peak and the manufacturing process, contamination of Printex-U with copper is unlikely.

##### 2.1.S.3.1 Elucidation of Structure and Other Characteristics

Not Applicable

##### 2.1.S.3.2 Impurities

No heavy metals

***2.1.S.4 CONTROL OF SUBSTANCE***

Table 2: Characteristics of Printex-U

| **Characteristics** | **Printex-U** |
| --- | --- |
| Primary particle size | 25 nm |
| Mean size of aggregates | < 200 nm |
| Arrangement | Onion-like |

##### 2.1.S.4.1 Specification

Not Applicable

##### 2.1.S.4.2 Analytical Procedures

Printex-U was characterized by the technicians of the department of Catalysis Engineering of the Technical University in Delft.

Transmission Electron Microscopy

Transmission electron microscopy (TEM) (figure 5) is a [microscopy](http://en.wikipedia.org/wiki/Microscope) technique whereby a beam of [electrons](http://en.wikipedia.org/wiki/Electron) is transmitted through an ultra thin specimen, interacting with the specimen as it passes through. An image is formed from the interaction of the electrons transmitted through the specimen; the image is magnified and [focused](http://en.wikipedia.org/wiki/Focus_(optics)) onto an imaging device, such as a [fluorescent](http://en.wikipedia.org/wiki/Fluorescent) screen, on a layer of [photographic film](http://en.wikipedia.org/wiki/Photographic_film), or to be detected by a sensor such as a charge-coupled device ([CCD) camera](http://en.wikipedia.org/wiki/CCD_camera).

TEMs are capable of imaging at a significantly higher [resolution](http://en.wikipedia.org/wiki/Optical_resolution) than light microscopes. Alternate modes of use allow for the TEM to observe modulations in chemical identity, crystal orientation, electronic structure and sample induced electron phase shift as well as the regular absorption based imaging.

Measurements were performed using a FEI Tecnai TF20 electron microscope with a field emission gun as the source of electrons operated at 200 kV. Samples were mounted on Quantifoil® carbon polymer supported on a copper grid by placing a few droplets of a suspension of sample in NaCl solution on the grid, followed by drying at ambient conditions.

[
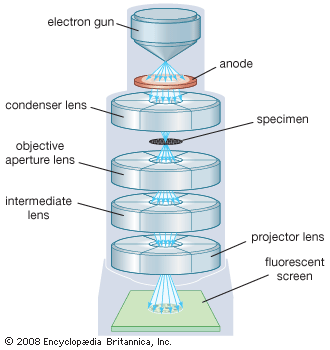
](http://arcticboy.arcticboy.com/view2.php?q=Transmission%20Electron%20Microscope%20Pictures&url=http://media-2.web.britannica.com/eb-media/90/113690-004-CB552E7F.gif)

Figure 5: Schematic figure of a Transmission Electron Microscope

##### 2.1.S.4.3 Validation of Analytical Procedures.

See Appendix III for the most recent maintenance certificate.

##### 2.1.S.4.4 Batch Analyses

After delivery of the product to the Academic Medical Centre it was stored in our Institutional Pharmacy according to GCP (Good Clinical Practice) and GMP (Good Manufacturing Practice) regulations.

Batch Analyses were performed by the department of Catalysis Engineering of the Technical University in Delft, the Netherlands.

***2.1.S.4.5 Table 3:*** Justification of specification

| *Attribute* | *Method* | *Acceptance criteria* | *Printex-U* |
| --- | --- | --- | --- |
| *Appearance* | *Visual observation* | *Black powder* | *Black, odourless powder* |
| *Melting point* | *PhEur* | *> 500 °C* | *> 3000 °C* |
| *Purity*  *- Heavy metals* | *Element analysis by* Energy-dispersive X-ray spectroscopy | *< 2%* | *Not detectable*  *(tested by the TU-Delft)* |
| *Water solubility* |  | *in soluble* | *in soluble* |
| *pH* |  | *~ 7 (50 g/l, 20°C)* | *< 7 (50 g/l, 20°C)* |
| *Flammability* | *VDI 2263* | *> 30 s* | *> 45 s* |
| *Ignition Temperature* | *VDI 2263 (BAM-furnace)* | *> 200 °C* | *> 300 °C* |
| *Autoinflammability* | *IMDG-Code with 1L sample* | *> 140 °C* | *> 140 °C* |
| *Lower explosion limit* | *VDI 2263* | *Dust 50 g/m^3^* | *Dust 50 g/m^3^* |
| *Upper explosion limit* |  | *Not determined* |  |
| *Max absolute explosive pressure* | *VDI 2263* | *10 bar* | *10 bar* |
| *Vapour pressure* |  | *Not applicable* |  |
| *Density* | *At 20 °C* | *1,7 – 1,9 g/cm^3^* | *1,7 – 1,9 g/cm^3^* |
| *Bulk density* |  | *50 – 250 kg/m^3^* | *50 – 250 kg/m^3^* |
| *Viscosity, dynamic* |  | *Not applicable* |  |

See also Material Safety Data Sheet, Appendix I

#### 2.1.S.5 REFERENCE STANDARDS OR MATERIALS

Not Applicable

#### 2.1.S.6 CONTAINER CLOSURE SYSTEM

Due to the risk of soiling it is very important that all handling be confined to closed systems. Due to its low bulk density and relatively low flow ability, Carbon Black powder is not shipped in rigid containers or silos, but for our purpose in FIBCs (Flexible Intermediate Bulk Containers).

#### 2.1.S.7 STABILITY

See Material Safety Data Sheet, Appendix I.

Carbon Black is very stable. We do not expect any disintegration of Printex-U at room temperature.

A temperature above 250^o^ Celsius should be avoided regarding the ignition temperature of 300^o^ Celsius.

In the case of fire, decomposition products are: Carbon monoxide, carbon dioxide (CO2), organic products of decomposition, sulphoxides

Carbon black cannot easily be caused to explode and therefore there is no danger in practical use.

**2.1.P PRODUCT**

#### 2.1.P.1 DESCRIPTION AND COMPOSITION OF THE PRODUCT

The product is an isotonic suspension of Printex-U and Sodium Chloride in sterile water. See chapter 2.1.P.2.2. and 2.3.6 (Clinical Pharmacokinetics) for dosages.

***2.1.P.2 PHARMACEUTICAL DEVELOPMENT***

During the preparation of this clinical trial we also tested Printex-F-α, another carbon black product from Evonik. We found Printex-F-α to be less comparable with carbon nanoparticles in diesel exhaust according to shape and arrangement.

***2.1.P.2.1 Components of the Product***

Printex-U; Sodium Chloride; Aqua dest.

See appendix IV for information on composition and dilution.

***2.1.P.2.2 Product***

The product will be manufactured in three different concentrations:

- 10 μg Printex-U in 10 ml saline

- 50 μg Printex-U in 10 ml saline

- 100 μg Printex-U in 10 ml saline

***2.1.P.2.3 Manufacturing Process Development***

***2.1.P.2.4 Container Closure System***

To prevent adherence of Printex-U to plastic, we choose glass bottles type I as primary packaging with omniflex rubber stopper as closure system.

***2.1.P.2.5 Microbiological Attributes***

- Because of possible interference of particles with the Limulus Amebocyte Lysate Assay, it was only performed on the lowest concentration of the test-batch, two months after preparation.

- Analysis by TEM was done in 15 different samples with a maximum of 6 months after preparation.

***2.1.P.2.6 Compatibility***

Suspension

***2.1.P.3 MANUFACTURE***

##### 2.1.P.3.1 Manufacturer(s)

Institutional Pharmacy of the University of Utrecht

Contact Drs. W. Meulenhoff

Adress Apotheek UMC Utrecht
Huispostnummer D 00.218
Postbus 85500
3508 GA Utrecht

**Faxnummer** 030 251 67 56

Email Address W.Meulenhoff@umcutrecht.nl

##### 2.1.P.3.2 Batch Formula

##### 2.1.P.3.3 Description of Manufacturing Process and Process Controls

Manufacturing Process

The product will be manufactured per patient under sterile conditions in a laminar airflow cabinet. The Printex-U powder will be accurately weighed and mixed with pulverized NaCl. A precisely weighed amount of this mixture will be suspended in sterile water. The product will be filled up to an isotone mixture of 10 ml before sterilization (121^o^ Celsius, 15 min). See appendix IV for the production protocol, product labels and appendix V for prints of the sterilization process.

Process Controls

A second control of weighing Printex-U powder and filling up to a standard volume will be performed as process controls.

##### 2.1.P.3.4 Controls of Critical Steps and Intermediates

##### Sterilization will be performed in a validated autoclave. Prints of the sterilization process are available in appendix V. See chapter 2.1.P.3.3.

##### 2.1.P.3.5 Process Validation and/or Evaluation

#### See chapter 2.1.P.3.4

#### 2.1.P.4 CONTROL OF EXCIPIENTS

Sodium Chloride is of PhEur quality

##### 2.1.P.4.1 Specifications

Not Applicable

##### 2.1.P.4.2 Analytical Procedures

See chapter 2.1.P.5.2

***2.1.P.4.3 Validation of Analytical procedures***

See chapter 2.1.P.5.3

***2.1.P.4.4 Justification of Specifications***

Not Applicable

##### 2.1.P.4.5 Excipients of Human or Animal Origin

*Not Applicable*

##### 2.1.P.4.6 Novel Excipients

*Not Applicable.*

#### 2.1.P.5 CONTROL OF PRODUCT

2.1.P.5.1 Specifications (s)

##### Table 4: specifications for Printex-U- suspension in saline

| ***Test Item*** | ***Method*** | ***Acceptance Criteria*** |
| --- | --- | --- |
| *Description* | *Visual*  *observation* | *Grey suspension* |
| *Primary particle size* | *Transmission Electron Microscopy (TEM)* | *< 100nm* |
| *Ratio agglomerates < 100nm vs > 100nm* | *Nanoparticle Tracking Analysis (NTA)* | *> 50% agglomerates/ particles < 100nm* |
| *Purity* | *TEM/EDX* | *99% carbon particles, description of the other 1 %* |
| *Arrangement* | *TEM* | *Onion-like* |
| *Contamination* | *TEM* | *No heavy metals* |
| *Microbiologic contamination* | *LAL-test (according to Annex 10)* | *< 0.1 Eu/ml* |

***2.1.P.5.2 Analytical Procedures***

We analyzed a test batch of the 3 concentrations of the product and matching placebo by Transmission Electron Microscopy, Energy-Dispersive X-ray spectroscopy, Nanoparticle Tracking Analysis, Dynamic Light Scatter (DLS), asymmetric-flow field-flow fractionation (AF4), and Limulus Amebocyte Lysate Assay (LAL-test). Unfortunately, the concentration of our samples is below the detection limit of the DLS and the AF4.

Batch analyses were performed by the department of Catalysis Engineering of the Technical University in Delft, the Netherlands.

Transmission Electron Microscopy (TEM) & Energy-dispersive X-ray spectroscopy (EDX)

See chapter 2.1.S.4.2

Nanoparticle Tracking Analysis (NTA)

Nanoparticle tracking analysis is a method for visualizing and analyzing particles in liquids that relates the rate of [Brownian motion](http://en.wikipedia.org/wiki/Brownian_motion) to particle size. NTA allows the determination of a size distribution profile of small particles (10-1000nm) in liquid suspension. The light scattered by the particles is captured using a charge coupled device ([CCD](http://en.wikipedia.org/wiki/Charge-coupled_device)) or electron multiplying (EM)-CCD camera and the motion of each particle is tracked from frame to frame. This rate of particle movement is related to a sphere equivalent [hydrodynamic](http://en.wikipedia.org/wiki/Hydrodynamic) radius as calculated through the Stokes–Einstein equation. The technique calculates particle size on a particle-by particle basis.

Limulus Amebocyte Lysate Assay:

Limulus amebocyte lysate (LAL) is an aqueous extract of blood cells ([amoebocytes](http://en.wikipedia.org/wiki/Amoebocyte)) from the [horseshoe crab](http://en.wikipedia.org/wiki/Horseshoe_crab), *Limulus polyphemus* (figure 6). LAL reacts with bacterial [endotoxin](http://en.wikipedia.org/wiki/Endotoxin) or [lipopolysaccharide](http://en.wikipedia.org/wiki/Lipopolysaccharide) (LPS), which is a membrane component of [Gram negative bacteria](http://en.wikipedia.org/wiki/Gram_negative_bacteria). This reaction is the basis of the LAL test, which is used for the detection and quantification of bacterial endotoxins in a product.


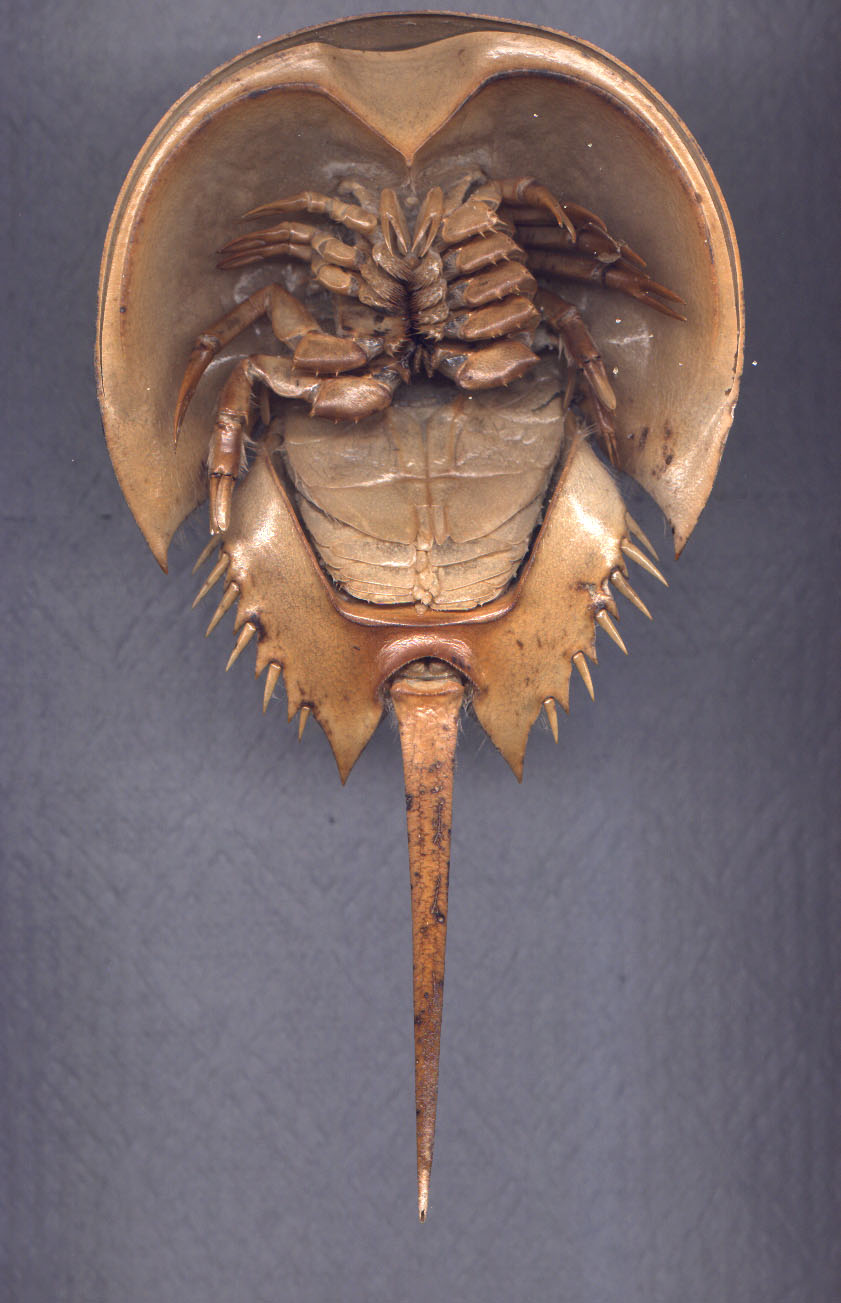


Figure 6: Horseshoe crab, *Limulus polyphemus.*

##### 2.1.P.5.3 Validation of Analytical Procedures

Not applicable

##### 2.1.P.5.4 Batch Analyses

##### Transmission electron microscopy:

Printex particles in the suspension had an onion-like arrangement and a primary particle size of 25 nm. We observed some dust particles in our printex-suspension as well as in our placebo samples. This contamination accounts for less than 5% of the total amount of particles and is probably a result of the unsterile conditions during preparation of the measurement. We also noticed NaCl-crystals in printex-suspension and placebo samples in an equal amount. None of the 15 samples we tested showed bacterial contamination as measured by TEM.

Energy-dispersive X-ray spectroscopy (EDX):

We observed no contamination with heavy metals in our test-batch.

Nanoparticle tracking analysis:

Particles and clusters in the lowest concentration are smaller then 150 nm (appendix VI). In the highest concentration the size of agglomerates is higher, with a mean size of 380nm (appendix VII).

Limulus Amebocyte Lysate Assay:

Printex in water: 20 mcg in 10 ml = <0,0100 EU/ml endotoxin.

##### 2.1.P.5.5 Characterization of Impurities

Analyses concerning the purity of the product were performed by the technicians of the Technical University of Delft.

Analyses concerning size distribution were performed by the technicians of Leiden University. See appendices V and VI for size distributions (NTA) of the product in the lowest and the highest concentration.

##### 2.1.P.5.6 Justification of Specification(s)

Table 5: justification of specifications for *Printex-U/ NaCl/ Aqua dest- suspension*

| ***Test Item*** | ***Method*** | ***Acceptance Criteria*** | ***PrintexU-suspension in saline*** |
| --- | --- | --- | --- |
| *Description* | *Visual*  *observation* | *Grey suspension* | Grey suspension |
| *Primary particle size* | *Transmission Electron Microscopy (TEM)* | *< 100 nm* | 25 nm |
| *Size distribution* | *NTA* | *> 50% agglomerates/ particles < 100 nm* | > 50% agglomerates/ particles < 100 nm |
| *Purity* | *TEM/EDX* | *99% carbon particles, description of the other 1 %* | > 99% carbon particles. Sporadically dust particle |
| *Arrangement* | *TEM* | *Onion-like* | Onion-like |
| *Microbiologic contamination* | *LAL-test* | *< 0.1 Eu/ml* |  |

In preparation of the current study we analyzed different carbon black products (Printex-F-α from Evonik and Carbon Nanopowder from Nanostructured & Amorphous Materials) regarding primary particles size, arrangement, and purity. We searched for a particle with similar size, shape and arrangement like carbon particles in regular soot because we want to develop a model mimicking normal exposure to air pollution.

#### 2.1.P.6 REFERENCE STANDARDS

Not Applicable

#### 2.1.P.7 CONTAINER CLOSURE SYSTEM

#### Glass bottles type I with omniflex rubber stopper as closure system.

#### 2.1.P.8 STABILITY

The exploratory stability data for the product show an increase in particle agglomerates in time. More specifically, when analyzed one week after manufacturing the main part of the particles were smaller than 100 nm but after one month the amount of clusters with a larger size was increased. Therefore we decided to make a new sample for each study participant at a maximum of one week before administration.

## Non-clinical pharmacology, pharmacokinetics and toxicology

- - 1. Test Materials used in toxicity studies

##### Acute toxicity

The median Lethal Dose for 50% of subjects (LD50) acute toxicity rating, oral ingestion by a rat, is above 10,000 mg/kg. Carbon Black application on intact skin and on the eye of a rabbit have no irritating effect (Appendix I).

##### Chronic toxicity

In the early 1990s, extended long-term inhalation studies with rats showed lung fibrosis and tumor development in case of extremely high dosages of Carbon Black particles. Mouse and hamster do not develop lung tumor under similar testing conditions (Appendix I).

In October 1995, the International Agency for Research on Cancer (IARC) reviewed the evaluation of Carbon Black and recommended an amendment to the rating. As a result, the evaluation of epidemiological data (“inadequate evidence”) still applies, though the overall rating was amended from Category 3 to Category 2B (“possible human carcinogen”) based on the long-term inhalation study made on rats under conditions of lung overload. The IARC evaluation in 2006 resulted in no changes of this rating^1^.

Based on findings by the National Toxicology Program (NTP/USA) as well as European and American legislation regarding chemicals (OSHA), Rubber Blacks and Carbon Black Pigments do not exhibit a mutagenic, teratogenic or carcinogenic potential (http://ntp.niehs.nih.gov).

##### Ecotoxicology

Carbon Black is an inorganic water insoluble substance. For this reason its bioavailability for aquatic organisms is very low. In acute tests according to OECD (Organisation for Economic Cooperation and Development) test guidelines with fish, daphnia and algae nominal concentrations of 1000 mg/l showed no effects. Based on the physicochemical and acute toxicological data no chronic effects and no bioaccumulation are to be expected in aquatic organisms. The general guidelines for the examination of the biodegradability of substances (OECD, EEC-guidelines) can be used only for organic substances. Carbon Black is an inert inorganic substance with the structural formula “C” and is not biodegradable by microorganisms. The German commission for the evaluation of water polluting substances has classified Carbon Black as a “not water endangering” substance (KBwS-No: 1742).

- - 1. Integrated assessment of the data package

Not Applicable

- - 1. List of studies Conducted & References

Not Applicable

- - 1. GLP statement and bioanalytical methods

Not Applicable

## CLINICAL DATA

- - 1. Clinical pharmacology
       1. Brief summary
    2. Clinical pharmacokinetics

Vialing the right dosages was performed according to Good Manufacturing Practice (GMP) regulations by the Institutional Pharmacy of the University in Utrecht, the Netherlands. Suspensions were made in sterile saline.

Volunteers will be given escalating dosages of 10 μg, 50 μg, and 100 μg. Dosages were calculated according to the European Medicines Agency (EMA) First-In-Man (FIM) guidelines based on the No Observed Adverse Effect Level (NOAEL) in non-clinical safety studies adjusted with allometric factors. These dosages are comparable with the mean exposure concentration of PM 2.5 (particles smaller than 2.5 µm) during public fireworks at New Year’s Day at the first hour of the year, as measured by the Dutch National Air Quality Monitoring Network (<http://www.lml.rivm.nl/>)^2^.

The mean peak concentration of the first hour of 2009 and 2010 is 1290 μg per cubic metre^2^. An adult subject exchanges 0.3 m^3^ of air per hour which compares to 387 micrograms of particulate matter. Because of the small size (<10 um), approximately 50% remain behind in the lung corresponding with 193.5 μg of particulate matter. When divided by five, there is an estimated deposition of 38.7 μg per lung lobe.

- - 1. Human exposure

Air quality in Europe:

A clean air supply is essential to our own health and that of the environment. But since the industrial revolution, the quality of the air we breathe has deteriorated considerably. Rising industrial and energy production, the burning of fossil fuels and the dramatic rise in traffic all contribute to air pollution which can lead to serious health problems, like an increase in prevalence of asthma for example^3^.

Since the early 1970s, the issue of air quality is a major concern for the European Union. As the result of EU legislation, much progress has already been made in reducing air pollutants but still, more needs to be done at local, national, European and international level.

The EU is acting at many levels to reduce exposure to air pollution: through EC legislation, through work at international level to reduce cross-border pollution, through co-operation with sectors responsible for air pollution, through national, regional authorities, and through research.

The new [**Directive 2008/50/EC**](http://eur-lex.europa.eu/LexUriServ/LexUriServ.do?uri=CELEX:32008L0050:EN:NOT) on ambient air quality and cleaner air for Europe was adopted by the Council and the European Parliament in May 2008, and entered into force on 11 June 2008. This Directive establishes health based standards and objectives for a number of pollutants in air.

Air quality in the Netherlands:

Measurements of the Dutch National Air Quality Monitoring Network demonstrate that the concentration of athmospheric pollutants measured in 2009 are not markedly different from those of previous years^2^.

There was no exceedance of the limit values for particulate matter (PM10), and the concentrations of heavy metals were below the lowest threshold.

In 2008, measurements for PM2.5 started, giving indicative results about the concentration levels in the Netherlands. The average PM2.5 concentration, in 2009 in the Netherlands, was 15 μg/m^3^ (Range: 13-21 μg/m^3^, European limit value: 25 μg/m^3^). Incidentally, exceeding of this limit value for PM2.5 occurred, mostly in the region Amsterdam/Haarlem.

Exposure to Carbon Black (Printex-U):

##### In decades of Carbon Black production and processing using a variety of methods, no significant hazardous effects have so far been registered.

On the basis of the data of the manufacturer, the product is not a hazardous substance as defined by the Chemicals Act or Hazardous Substance Ordinance in the currently valid versions

See Appendix I for MSDS sheet.

## Overall risk and benefit assessment

Carbon Black Nanoparticles (Printex-U)

Although Carbon Black Nanoparticles in its pure form was not studied in humans before, there are several studies investigating diluted diesel exhaust which is mainly composed of carbon particles. So far, challenge with diluted diesel exhaust did not induce clinical signs or symptoms^4-6^.

After our thorough analysis of the raw and final product we believe the quality is good and the product resembles the properties of nanoparticles seen in air pollution.

Segmental instillation by bronchoscope

The protocol for segmental instillation by bronchoscope followed by a second bronchoscopy to investigate the local inflammatory response and activation of coagulation pathways has been widely published in the international literature^7, 8^. The most important discomfort experienced during a bronchoscopy is a dry cough and pain in the nose on introduction of the scope. This can be prevented by local anaesthesia of the mucosa with lidocaïne. In our own experience the procedure of both the bronchoscopy and bronchoalveolar lavage (BAL) or biopsy is tolerated very well by healthy non-smoking subjects as well as mild asthmatics.

In this protocol, nanoparticle exposure is limited to 1 subsegment of the right middle lobe or lingual. Therefore, the amount of nanoparticles administered and the change of inducing a general bronchus obstruction is far less compared to whole lung exposure.

Study design

For safety reasons, we will perform a dose-escalation study. After completion of every dosage group, an interim-analysis will be done on safety parameters, symptoms and white blood cell count in bronchoalveolar lavage fluid by an independent data safety monitoring board (DSMB). If any adverse event will occur, the previous dose will be regarded as maximum tolerable dose (MTD), and this dosage group will be completed up to 9 volunteers. Also, if there would be a mean difference of 50% or more in BAL leukocytes (control versus challenge lung), this dosage will be regarded as minimum effective dose (MED) for this model and this group will be completed up to 9 volunteers.

Management of possible effects/adverse events

During the study day, subjects will be accompanied by a physician and kept on observation in the hospital. Hemodynamic measures, oxygen saturation, and lung function will be measured regularly. In case of bronchoconstriction there will be inhalation medication available, ready to use. In case of more serious adverse events, subjects will be analysed, diagnosed, and treated by pulmonologists.

REFERENCES

1. Ward EM, Schulte PA, Straif K et al. Research recommendations for selected IARC-classified agents. Environ Health Perspect 2010;118(10):1355-1362.

2. Mooibroek D, Beijk R, Hoogerbrugge R. RIVM Jaaroverzicht Luchtkwaliteit 2009. 2010

3. von KS, Wolke G, Tuch T et al. Increased asthma medication use in association with ambient fine and ultrafine particles. Eur Respir J 2002;20(3):691-702.

4. Lucking AJ, Lundback M, Mills NL et al. Diesel exhaust inhalation increases thrombus formation in man. Eur Heart J 2008;29(24):3043-3051.

5. Mills NL, Tornqvist H, Robinson SD et al. Diesel exhaust inhalation causes vascular dysfunction and impaired endogenous fibrinolysis. Circulation 2005;112(25):3930-3936.

6. Salvi S, Blomberg A, Rudell B et al. Acute inflammatory responses in the airways and peripheral blood after short-term exposure to diesel exhaust in healthy human volunteers. Am J Respir Crit Care Med 1999;159(3):702-709.

7. Hoogerwerf JJ, de Vos AF, Bresser P et al. Lung inflammation induced by lipoteichoic acid or lipopolysaccharide in humans. Am J Respir Crit Care Med 2008;178(1):34-41.

8. O'Grady NP, Preas HL, Pugin J et al. Local inflammatory responses following bronchial endotoxin instillation in humans. Am J Respir Crit Care Med 2001;163(7):1591-1598.
