# Supplementary material for: Pulmonary challenge with carbon nanoparticles induces a dose-dependent increase in circulating leukocytes in healthy males
Source: BMC Pulm Med. 2017 Sep 6;17:121. doi: 10.1186/s12890-017-0463-x (PMC5588713; doi:10.1186/s12890-017-0463-x)
Supplement: Supplementary file 2 — Inflammation parameters in blood and BALF after challenge with placebo or 10 μg nanoparticles. Values are expressed as median with minimum and maximums values. Analysis was performed with non-parametric Mann - Whitney test, after correction for baseline values. Definition of abbreviations: NP: nanoparticles, CRP: C-reactive protein. Table S2. Inflammation parameters in blood and BALF after challenge with placebo or 50 μg nanoparticles. Values are expressed as median with minimum and maximum values. Analysis was performed with non-parametric Mann - Whitney test, after correction for baseline values. Definition of abbreviations: NP: nanoparticles, CRP: C-reactive protein. Table S3. Inflammation parameters in blood and BALF after challenge with 10 μg or 50 μg nanoparticles. Values are expressed as median with minimum and maximum values. Analysis was performed with non-parametric Mann - Whitney test, after correction for baseline values. Definition of abbreviations: NP: nanoparticles, CRP: C-reactive protein. Table S4. Inflammation parameters in blood and BALF after challenge with 10 μg or 100 μg nanoparticles. Values are expressed as median with minimum and maximum values. Analysis was performed with non-parametric Mann - Whitney test, after correction for baseline values. Definition of abbreviations: NP: nanoparticles, CRP: C-reactive protein. Table S5. Inflammation parameters in blood and BALF after challenge with 50 μg or 100 μg nanoparticles. Values are expressed as median with minimum and maximum values. Analysis was performed with non-parametric Mann - Whitney test, after correction for baseline values. Definition of abbreviations: NP: nanoparticles, CRP: C-reactive protein. (DOC 193 kb) [file 12890_2017_463_MOESM2_ESM.doc]

Additional file 2

Table S1: Inflammation parameters in blood and BALF after challenge with placebo or 10 µg nanoparticles.

| **Inflammation Parameters**  **Blood** | **Placebo**  **N = 9** | **10 µg NP**  **N = 4** | **P-value** |
| --- | --- | --- | --- |
| Leukocytes, Cells x 10^9^/L | 6.60  (4.8 – 8.5) | 6.05  (5.10 - 7.20) | 0.559 |
| Neutrophils, Cells x 10^9^/L | 4.35  (2.9 – 6.6) | 3.52  (2.64 – 5.81) | 0.559 |
| Lymphocytes, Cells x 10^9^/L | 1.76  (1.4 – 2.3) | 1.77  (0.86 – 2.17) | 0.070 |
| Monocytes, Cells x 10^9^/L | 0.52  (0.3 – 0.6) | 0.45  (0.43 – 0.61) | 1.000 |
| Eosinophils, Cells x 10^9^/L | 0.11  (0.1 – 0.3) | 0.05  (0.04 – 0.15) | 1.000 |
| Basophils, Cells x 10^9^/L | 0.03  (0.0 – 0.1) | 0.04  (0.02 – 0.05) | 1.000 |
| CRP, mg/L | 0.50  (0.3 – 7.4) | 0.40  (0.30 - 2.30) | 1.000 |
| **Inflammation Parameters**  **BALF** | **Placebo**  **N = 8** | **10 µg NP**  **N = 4** | **P-value** |
| Leukocytes, Cells x 10^4^/ml | 9.47  (6.5 – 36.5) | 8.79  (4.62 – 11.84) | 1.000 |
| Neutrophils, Cells x 10^4^/ml | 0.33  (0.1 – 2.6) | 1.10  (0.08 - 6.82) | 0.545 |
| Lymphocytes, Cells x 10^4^/ml | 0.31  (0.1 – 0.7) | 0.68  (0.56 – 0.88) | 0.545 |
| Monocytes, Cells x 10^4^/ml | - | - | - |
| Eosinophils, Cells x 10^4^/ml | 0.03  (0.0 – 0.3) | 0.00  (0.00 – 0.05) | 0.576 |
| Macrophages, Cells x 10^4^/ml | 8.72  (6.0 – 33.1) | 5.20  (3.63 – 8.03) | 0.545 |
| Mast cells, Cells x 10^4^/ml | 0.00  (0.0 – 0.0) | 0.00  (0.00 – 0.01) | 1.000 |
| Plasma cells, Cells x 10^4^/ml | - | 0.00  (0.00 – 0.02) | 0.333 |
| ECP, ng/ml | 0.65  (0.3 – 2.3) | 0.62  (0.16 – 2.00) | 0.545 |
| MPO, ng/ml | 13.91  (5.1 – 43.3) | 15.12  (2.11 - 56.07) | 1.000 |
| GRO-α, ng/ml | 0.60  (0.3 – 0.9) | 0.69  (0.48 – 0.85) | 1.000 |
| IL-6, pg/ml | 5.39  (1.0 – 57.0) | 49.17  (0.75 - 247.82) | 1.000 |
| IL-8, ng/ml | 0.03  (0.0 – 0.5) | 0.26  (0.01 – 0.78) | 1.000 |
| IL-17, pg/ml | 1.60  (1.6 – 1.6) | 1.60  (1.60 – 1.60) | - |
| CXCL-10, ng/ml | 0.66  (0.1 – 5.6) | 1.65  (1.19 – 3.37) | 1.000 |
| MCP-1, ng/ml | 0.02  (0.0 – 0.1) | 0.04  (0.01 – 0.15) | 1.000 |
| MIP-1α, pg/ml | 0.15  (0.2 – 8.5) | 12.68  (0.15 - 70.89) | 1.000 |
| MIP-1β, ng/ml | 0.06  (0.0 – 1.0) | 1.60  (0.01 - 5.37) | 1.000 |
| TNF-α, pg/ml | 0.60  (0.6 – 1.5) | 3.92  (0.60 - 11.29) | 0.236 |
| VEGF, ng/ml | 0.78  (0.0 – 1.7) | 1.18  (0.97 – 3.08) | 1.000 |

Values are expressed as median with minimum and maximum values. Analysis was performed with non-parametric Mann - Whitney test, after correction for baseline values.

*Definition of abbreviations:* *NP: nanoparticles, CRP: C-reactive protein*.

Table S2: Inflammation parameters in blood and BALF after challenge with placebo or 50 µg nanoparticles

| **Inflammation Parameters**  **Blood** | **Placebo**  **N = 9** | **50 µg NP**  **N = 4** | **P-value** |
| --- | --- | --- | --- |
| Leukocytes, Cells x 10^9^/L | 6.60  (4.8 – 8.5) | 7.50  (5.20 – 10.40) | 1.000 |
| Neutrophils, Cells x 10^9^/L | 4.35  (2.9 – 6.6) | 5.16  (2.84 – 7.79) | 1.000 |
| Lymphocytes, Cells x 10^9^/L | 1.76  (1.4 – 2.3) | 1.78  (1.37 – 2.19) | 0.559 |
| Monocytes, Cells x 10^9^/L | 0.52  (0.3 – 0.6) | 0.54  (0.37 – 0.76) | 1.000 |
| Eosinophils, Cells x 10^9^/L | 0.11  (0.1 – 0.3) | 0.07  (0.02 – 0.14) | 0.266 |
| Basophils, Cells x 10^9^/L | 0.03  (0.0 – 0.1) | 0.03  (0.02 – 0.04) | 0.497 |
| CRP, mg/L | 0.50  (0.3 – 7.4) | 0.35  (0.30 - 2.70) | 1.000 |
| **Inflammation Parameters**  **BALF** | **Placebo**  **N = 8** | **50 µg NP**  **N = 4** | **P-value** |
| Leukocytes, Cells x 10^4^/ml | 9.47  (6.5 – 36.5) | 9.17  (7.90 – 12.04) | 1.000 |
| Neutrophils, Cells x 10^4^/ml | 0.33  (0.1 – 2.6) | 0.23  (0.09 – 0.48) | 0.545 |
| Lymphocytes, Cells x 10^4^/ml | 0.31  (0.1 – 0.7) | 0.50  (0.32 – 0.65) | 0.545 |
| Monocytes, Cells x 10^4^/ml | - | - | - |
| Eosinophils, Cells x 10^4^/ml | 0.03  (0.0 – 0.3) | 0.02  (0.00 - 0.04) | 0.576 |
| Macrophages, Cells x 10^4^/ml | 8.72  (6.0 – 33.1) | 8.34  (7.36 – 11.12) | 0.545 |
| Mast cells, Cells x 10^4^/ml | 0.00  (0.0 – 0.0) | 0.00  (0.00 – 0.00) | 1.000 |
| Plasma cells, Cells x 10^4^/ml | - | - | - |
| ECP, ng/ml | 0.65  (0.3 – 2.3) | 0.55  (0.27 – 0.97) | 0.545 |
| MPO, ng/ml | 13.91  (5.1 – 43.3) | 9.04  (5.02 – 20.99) | 1.000 |
| GRO-α, ng/ml | 0.60  (0.3 – 0.9) | 0.55  (0.43 – 0.95) | 1.000 |
| IL-6, pg/ml | 5.39  (1.0 – 57.0) | 0.77  (0.35 – 2.30) | 0.545 |
| IL-8, ng/ml | 0.03  (0.0 – 0.5) | 0.01  (0.01 – 0.02) | 1.000 |
| IL-17, pg/ml | 1.60  (1.6 – 1.6) | 1.60  (1.60 – 1.60) | - |
| CXCL-10, ng/ml | 0.66  (0.1 – 5.6) | 0.77  (0.36 – 1.06) | 0.061 |
| MCP-1, ng/ml | 0.02  (0.0 – 0.1) | 0.00  (0.00 – 0.00) | 0.545 |
| MIP-1α, pg/ml | 0.15  (0.2 – 8.5) | 0.15  (0.15 – 0.15) | 0.491 |
| MIP-1β, ng/ml | 0.06  (0.0 – 1.0) | 0.01  (0.01 – 0.04) | 0.545 |
| TNF-α, pg/ml | 0.60  (0.6 – 1.5) | 0.60  (0.60 – 0.60) | 1.000 |
| VEGF, ng/ml | 0.78  (0.0 – 1.7) | 0.49  (0.32 – 1.52) | 1.000 |

Values are expressed as median with minimum and maximum values. Analysis was performed with non-parametric Mann - Whitney test, after correction for baseline values.

*Definition of abbreviations:* *NP: nanoparticles, CRP: C-reactive protein*.

Table S3: Inflammation parameters in blood and BALF after challenge with 10 µg or 50 µg nanoparticles.

| **Inflammation Parameters**  **Blood** | **10 µg NP**  **N = 4** | **50 µg NP**  **N = 4** | **P-value** |
| --- | --- | --- | --- |
| Leukocytes, Cells x 10^9^/L | 6.05  (5.10 - 7.20) | 7.50  (5.20 – 10.40) | 1.000 |
| Neutrophils, Cells x 10^9^/L | 3.52  (2.64 – 5.81) | 5.16  (2.84 – 7.79) | 0.486 |
| Lymphocytes, Cells x 10^9^/L | 1.77  (0.86 – 2.17) | 1.78  (1.37 – 2.19) | 0.486 |
| Monocytes, Cells x 10^9^/L | 0.45  (0.43 – 0.61) | 0.54  (0.37 – 0.76) | 1.000 |
| Eosinophils, Cells x 10^9^/L | 0.05  (0.04 – 0.15) | 0.07  (0.02 – 0.14) | 0.486 |
| Basophils, Cells x 10^9^/L | 0.04  (0.02 – 0.05) | 0.03  (0.02 – 0.04) | 1.000 |
| CRP, mg/L | 0.40  (0.30 - 2.30) | 0.35  (0.30 - 2.70) | - |
| **Inflammation Parameters**  **BALF** | **10 µg NP**  **N = 4** | **50 µg NP**  **N = 4** | **P-value** |
| Leukocytes, Cells x 10^4^/ml | 8.79  (4.62 – 11.84) | 9.17  (7.90 – 12.04) | 0.486 |
| Neutrophils, Cells x 10^4^/ml | 1.10  (0.08 - 6.82) | 0.23  (0.09 – 0.48) | 1.000 |
| Lymphocytes, Cells x 10^4^/ml | 0.68  (0.56 – 0.88) | 0.50  (0.32 – 0.65) | 0.486 |
| Monocytes, Cells x 10^4^/ml | - | - | - |
| Eosinophils, Cells x 10^4^/ml | 0.00  (0.00 – 0.05) | 0.02  (0.00 - 0.04) | 1,000 |
| Macrophages, Cells x 10^4^/ml | 5.20  (3.63 – 8.03) | 8.34  (7.36 – 11.12) | 0.486 |
| Mast cells, Cells x 10^4^/ml | 0.00  (0.00 – 0.01) | 0.00  (0.00 – 0.00) | 1.000 |
| Plasma cells, Cells x 10^4^/ml | 0.00  (0.00 – 0.02) | - | 1.000 |
| ECP, ng/ml | 0.62  (0.16 – 2.00) | 0.55  (0.27 – 0.97) | 0.486 |
| MPO, ng/ml | 15.12  (2.11 - 56.07) | 9.04  (5.02 – 20.99) | 1.000 |
| GRO-α, ng/ml | 0.69  (0.48 – 0.85) | 0.55  (0.43 – 0.95) | 0.486 |
| IL-6, pg/ml | 49.17  (0.75 - 247.82) | 0.77  (0.35 – 2.30) | 0.486 |
| IL-8, ng/ml | 0.26  (0.01 – 0.78) | 0.01  (0.01 – 0.02) | 0.486 |
| IL-17, pg/ml | 1.60  (1.60 – 1.60) | 1.60  (1.60 – 1.60) | - |
| CXCL-10, ng/ml | 1.65  (1.19 – 3.37) | 0.77  (0.36 – 1.06) | 1.000 |
| MCP-1, ng/ml | 0.04  (0.01 – 0.15) | 0.00  (0.00 – 0.00) | 0.486 |
| MIP-1α, pg/ml | 12.68  (0.15 - 70.89) | 0.15  (0.15 – 0.15) | 0.429 |
| MIP-1β, ng/ml | 1.60  (0.01 - 5.37) | 0.01  (0.01 – 0.04) | 0.486 |
| TNF-α, pg/ml | 3.92  (0.60 - 11.29) | 0.60  (0.60 – 0.60) | 0.429 |
| VEGF, ng/ml | 1.18  (0.97 – 3.08) | 0.49  (0.32 – 1.52) | 1.000 |

Values are expressed as median with minimum and maximum values. Analysis was performed with non-parametric Mann - Whitney test, after correction for baseline values.

*Definition of abbreviations:* *NP: nanoparticles, CRP: C-reactive protein*.

Table S4: Inflammation parameters in blood and BALF after challenge with 10 µg or 100 µg nanoparticles.

| **Inflammation Parameters**  **Blood** | **10 µg NP**  **N = 4** | **100 µg NP**  **N = 9** | **P-value** |
| --- | --- | --- | --- |
| Leukocytes, Cells x 10^9^/L | 6.05  (5.10 - 7.20) | 8.70  (5.60 – 12.40) | **0.050** |
| Neutrophils, Cells x 10^9^/L | 3.52  (2.64 – 5.81) | 6.15  (3.35 – 9.69) | 0.148 |
| Lymphocytes, Cells x 10^9^/L | 1.77  (0.86 – 2.17) | 1.77  (1.49 – 2.48) | **0.050** |
| Monocytes, Cells x 10^9^/L | 0.45  (0.43 – 0.61) | 0.59  (0.27 – 0.90) | 0.076 |
| Eosinophils, Cells x 10^9^/L | 0.05  (0.04 – 0.15) | 0.07  (0.02 – 0.39) | 0.503 |
| Basophils, Cells x 10^9^/L | 0.04  (0.02 – 0.05) | 0.02  (0.01 – 0.05) | 0.604 |
| CRP, mg/L | 0.40  (0.30 - 2.30) | 0.40  (0.30 - 3.50) | 0.503 |
| **Inflammation Parameters**  **BALF** | **10 µg NP**  **N = 4** | **100 µg NP**  **N = 9** | **P-value** |
| Leukocytes, Cells x 10^4^/ml | 8.79  (4.62 – 11.84) | 11.63  (5.69 - 18.72) | 1.000 |
| Neutrophils, Cells x 10^4^/ml | 1.10  (0.08 - 6.82) | 0.58  (0.11 - 3.84) | 0.604 |
| Lymphocytes, Cells x 10^4^/ml | 0.68  (0.56 – 0.88) | 0.40  (0.14 - 1.65) | 1.000 |
| Monocytes, Cells x 10^4^/ml | - | - | - |
| Eosinophils, Cells x 10^4^/ml | 0.00  (0.00 – 0.05) | 0.00  (0.00 – 0.09) | 0.604 |
| Macrophages, Cells x 10^4^/ml | 5.20  (3.63 – 8.03) | 9.04  (4.96 – 16.94) | 0.710 |
| Mast cells, Cells x 10^4^/ml | 0.00  (0.00 – 0.01) | 0.00  (0.00 – 0.00) | 0.503 |
| Plasma cells, Cells x 10^4^/ml | 0.00  (0.00 – 0.02) | 0.00  (0.00 – 0.00) | 1.000 |
| ECP, ng/ml | 0.62  (0.16 – 2.00) | 0.76  (0.10 – 1.87) | 0.940 |
| MPO, ng/ml | 15.12  (2.11 - 56.07) | 14.28  (6.28 – 35.70) | 1.000 |
| GRO-α, ng/ml | 0.69  (0.48 – 0.85) | 0.49  (0.24 - 1.47) | 0.503 |
| IL-6, pg/ml | 49.17  (0.75 - 247.82) | 25.57  (0.20 – 124.01) | 0.503 |
| IL-8, ng/ml | 0.26  (0.01 – 0.78) | 0.13  (0.01 - 2.17) | 0.503 |
| IL-17, pg/ml | 1.60  (1.60 – 1.60) | 1.60  (1.60 – 1.60) | 0.825 |
| CXCL-10, ng/ml | 1.65  (1.19 – 3.37) | 1.02  (0.11 - 5.31) | 0.710 |
| MCP-1, ng/ml | 0.04  (0.01 – 0.15) | 0.02  (0.00 – 0.83) | 0.604 |
| MIP-1α, pg/ml | 12.68  (0.15 - 70.89) | 3.76  (0.15 – 31.14) | 0.414 |
| MIP-1β, ng/ml | 1.60  (0.01 - 5.37) | 0.48  (0.01 - 7.10) | 0.330 |
| TNF-α, pg/ml | 3.92  (0.60 - 11.29) | 1.15  (0.60 - 15.11) | 0.330 |
| VEGF, ng/ml | 1.18  (0.97 – 3.08) | 0.51  (0.15 - 1.74) | 0.414 |

Values are expressed as median with minimum and maximum values. Analysis was performed with non-parametric Mann - Whitney test, after correction for baseline values.

*Definition of abbreviations:* *NP: nanoparticles, CRP: C-reactive protein*.

Table S5: Inflammation parameters in blood and BALF after challenge with 50 µg or 100 µg nanoparticles.

| **Inflammation Parameters**  **Blood** | **50 µg NP**  **N = 4** | **100 µg NP**  **N = 9** | **P-value** |
| --- | --- | --- | --- |
| Leukocytes, Cells x 10^9^/L | 7.50  (5.20 – 10.40) | 8.70  (5.60 – 12.40) | 0.710 |
| Neutrophils, Cells x 10^9^/L | 5.16  (2.84 – 7.79) | 6.15  (3.35 – 9.69) | 0.825 |
| Lymphocytes, Cells x 10^9^/L | 1.78  (1.37 – 2.19) | 1.77  (1.49 – 2.48) | 0.503 |
| Monocytes, Cells x 10^9^/L | 0.54  (0.37 – 0.76) | 0.59  (0.27 – 0.90) | 0.330 |
| Eosinophils, Cells x 10^9^/L | 0.07  (0.02 – 0.14) | 0.07  (0.02 – 0.39) | 0.604 |
| Basophils, Cells x 10^9^/L | 0.03  (0.02 – 0.04) | 0.02  (0.01 – 0.05) | 0.260 |
| CRP, mg/L | 0.35  (0.30 - 2.70) | 0.40  (0.30 - 3.50) | 0.604 |
| **Inflammation Parameters**  **BALF** | **50 µg NP**  **N = 4** | **100 µg NP**  **N = 9** | **P-value** |
| Leukocytes, Cells x 10^4^/ml | 9.17  (7.90 – 12.04) | 11.63  (5.69 - 18.72) | 0.330 |
| Neutrophils, Cells x 10^4^/ml | 0.23  (0.09 – 0.48) | 0.58  (0.11 - 3.84) | 0.825 |
| Lymphocytes, Cells x 10^4^/ml | 0.50  (0.32 – 0.65) | 0.40  (0.14 - 1.65) | 0.414 |
| Monocytes, Cells x 10^4^/ml | - | - | 0.825 |
| Eosinophils, Cells x 10^4^/ml | 0.02  (0.00 - 0.04) | 0.00  (0.00 – 0.09) | 0.414 |
| Macrophages, Cells x 10^4^/ml | 8.34  (7.36 – 11.12) | 9.04  (4.96 – 16.94) | 0.503 |
| Mast cells, Cells x 10^4^/ml | 0.00  (0.00 – 0.00) | 0.00  (0.00 – 0.00) | 1.000 |
| Plasma cells, Cells x 10^4^/ml | - | 0.00  (0.00 – 0.00) | 1.000 |
| ECP, ng/ml | 0.55  (0.27 – 0.97) | 0.76  (0.10 – 1.87) | 0.710 |
| MPO, ng/ml | 9.04  (5.02 – 20.99) | 14.28  (6.28 – 35.70) | 1.000 |
| GRO-α, ng/ml | 0.55  (0.43 – 0.95) | 0.49  (0.24 - 1.47) | 0.330 |
| IL-6, pg/ml | 0.77  (0.35 – 2.30) | 25.57  (0.20 – 124.01) | 0.604 |
| IL-8, ng/ml | 0.01  (0.01 – 0.02) | 0.13  (0.01 - 2.17) | 0.604 |
| IL-17, pg/ml | 1.60  (1.60 – 1.60) | 1.60  (1.60 – 1.60) | 0.825 |
| CXCL-10, ng/ml | 0.77  (0.36 – 1.06) | 1.02  (0.11 - 5.31) | 1.000 |
| MCP-1, ng/ml | 0.00  (0.00 – 0.00) | 0.02  (0.00 – 0.83) | 0.825 |
| MIP-1α, pg/ml | 0.15  (0.15 – 0.15) | 3.76  (0.15 – 31.14) | 0.825 |
| MIP-1β, ng/ml | 0.01  (0.01 – 0.04) | 0.48  (0.01 - 7.10) | 1.000 |
| TNF-α, pg/ml | 0.60  (0.60 – 0.60) | 1.15  (0.60 - 15.11) | 0.825 |
| VEGF, ng/ml | 0.49  (0.32 – 1.52) | 0.51  (0.15 - 1.74) | 0.260 |

Values are expressed as median with minimum and maximum values. Analysis was performed with non-parametric Mann - Whitney test, after correction for baseline values.

*Definition of abbreviations:* *NP: nanoparticles, CRP: C-reactive protein*.
